# Association of Gestational Weight Gain With Infant Morbidity and Mortality in the United States

**DOI:** 10.1001/jamanetworkopen.2021.41498

**Published:** 2021-12-30

**Authors:** Lijun Wang, Xiaoyu Zhang, Tingting Chen, Jun Tao, Yanduo Gao, Li Cai, Huijun Chen, Chuanhua Yu

**Affiliations:** 1 Department of Epidemiology and Biostatistics, School of Public Health, Guangxi Medical University, Nanning, China; 2 School of Public Health, University of Hong Kong, Hong Kong, China; 3 Ultrasound Diagnosis Department, Hubei Maternal and Child Health Hospital, Wuhan, China; 4 Department of Maternal and Child Health, School of Public Health, Sun Yat-Sen University, Guangzhou, China; 5 Department of Gynaecology and Obstetrics, Zhongnan Hospital of Wuhan University, Wuhan, China; 6 Department of Epidemiology and Biostatistics, School of Health Sciences , Wuhan University, Wuhan, China

## Abstract

**Importance:**

The associations of gestational weight gain (GWG) with infant morbidity and mortality are unclear, and the existing recommendations for GWG have not been stratified by the severity of obesity.

**Objectives:**

To identify optimal GWG ranges associated with reduced risks of infant morbidity and mortality across maternal body mass index (BMI) categories.

**Design, Setting, and Participants:**

This retrospective cohort study used US nationwide, linked birth and infant death data between 2011 and 2015 to assess the associations of GWG in 2.0-kg groups with infant morbidity and mortality and identified optimal GWG ranges associated with reduced risks of both outcomes, using multivariable logistic regression models. Statistical analysis was performed from February 11 to October 14, 2021.

**Exposure:**

Gestational weight gain equivalent to 40 weeks.

**Main Outcomes and Measures:**

The 2 main outcomes were (1) significant morbidity of the newborn infant, defined as any presence of assisted ventilation, admission to the neonatal intensive care unit, surfactant therapy, antibiotic therapy, or seizures; and (2) infant mortality younger than 1 year of age (<1 hour, 1-23 hours, 1-6 days, 7-27 days, or 28-365 days after birth).

**Results:**

In this study of 15 759 945 mother-infant dyads, the mean (SD) age of the women was 28.1 (5.9) years. Women gained a mean (SD) of 14.1 (7.3) kg during pregnancy, and the mean (SD) GWG decreased with BMI categories (underweight, 15.7 [6.4] kg; normal weight, 15.4 [6.2] kg; overweight, 14.2 [7.4] kg; obesity class 1, 12.2 [8.0] kg; obesity class 2, 10.3 [8.4] kg; obesity class 3, 8.2 [9.2] kg; *P* < .001). A total of 8.8% of the newborns experienced significant morbidity, with the lowest prevalence among infants delivered by women in the normal weight BMI class (8.0%) and the highest among infants delivered by women with class 3 obesity (12.4%); 0.34% of infants died within 1 year of birth, with the lowest prevalence among infants delivered by women in the normal weight BMI class (0.28%) and the highest among infants delivered by women with class 3 obesity (0.58%). Optimal GWG ranges were 12.0 to less than 24.0 kg for underweight and normal weight women, 10.0 to less than 20.0 kg for overweight women, 8.0 to less than 16.0 kg for women with class 1 obesity, 6.0 to less than 16.0 kg for class 2 obesity, and 6.0 to less than 10.0 kg for class 3 obesity. The lower bounds of the optimal GWG ranges appeared to be higher than the existing recommendations for overweight women (10.0 vs 7.0 kg) and for those with class 1 (8.0 vs 5.0 kg), class 2 (6.0 vs 5.0 kg), and class 3 (6.0 vs 5.0 kg) obesity.

**Conclusions and Relevance:**

This study analyzed the associations of GWG with infant morbidity and mortality across BMI categories and found that inadequate GWG was associated with increased risks of adverse infant outcomes even for women with obesity. The results suggested that weight maintenance or weight loss should not be used as routine guidelines.

## Key Points

**Question:**

Is there an association between gestational weight gain (GWG) and adverse infant outcomes among women across body mass index (BMI) categories?

**Findings:**

In this cohort study of 15.8 million mother-infant dyads, infant morbidity and mortality risks showed U-shaped associations with GWG. Inadequate GWG and weight loss were associated with increased risks across BMI categories, but the effect sizes were smaller among women with obesity.

**Meaning:**

In this study, extreme GWG ranges were associated with increased risks of infant morbidity and mortality across BMI categories, suggesting that prenatal weight loss or weight maintenance should not be encouraged.

## Teaser

This cohort study uses US nationwide, linked birth and infant death data to identify optimal gestational weight gain ranges associated with reduced risks of infant morbidity and mortality across maternal body mass index categories.

## Introduction

Adverse pregnancy outcomes are associated with inappropriate gestational weight gain (GWG). Inadequate GWG increases the risks of preterm birth and being born small for gestational age.^1,2^ Excessive GWG is linked to pregnancy complications such as gestational hypertension and diabetes, cesarean delivery, postpartum weight retention and obesity in later life, and adverse outcomes to the offspring such as being born large for gestational age, macrosomia, and childhood obesity.^1,2,3^

The existing recommendations for GWG were issued by the US National Academy of Medicine (NAM) in 2009.^4^ The recommendations were based on previous studies on potential associations of GWG with maternal and child health, and provided a single range of GWG (5.0-9.0 kg) for women with obesity, owing to insufficient evidence stratified by the severity of obesity. In 2019, the Maternal Obesity and Childhood Outcomes Study Group in the LifeCycle Project (henceforth referred to as “LifeCycle Project”) conducted a meta-analysis of individual participant data of 196 670 women and suggested respective GWG ranges for class 1 (2.0-6.0 kg), class 2 (≤4.0 kg), and class 3 (≤6.0 kg) obesity.^2^ Apart from the LifeCycle Project, growing evidence also suggested lower levels of GWG than the NAM recommendations, or even weight loss, for obese women.^5,6,7,8^

In examining the associations between GWG and infant outcomes, NAM, the LifeCycle Project, and many other studies used intermediate outcomes, such as preterm and postterm birth, born small for gestational age, and born large for gestational age, as proxy markers of ultimate health outcomes—infant morbidity and mortality.^1,2,3,4^ By contrast, directly addressing infant morbidity and mortality offers more informative and reliable estimates, especially if the total effects are not fully reflected by intermediate outcomes.^4^ However, ultimate health outcomes of the infant associated with GWG are understudied, to our knowledge. Inconsistent results have been reported on the benefits and risks of low GWG and weight loss associated with infant mortality among women with obesity.^9,10,11,12,13,14^ Studies on infant morbidity were fewer and showed that GWG outside the recommended ranges was associated with increased risks of neonatal intensive care unit (NICU) admission, assisted ventilation, and seizures.^15,16,17^ Moreover, this limited evidence on the associations of GWG with infant morbidity and mortality was rarely stratified by maternal body mass index (BMI),^9,11,12,13,14,15^ especially by the severity of obesity.^11^

Infant morbidity and mortality are important public health issues. The infant mortality rate is used widely as an overall indicator of reproductive health for a population, and neonatal anomalies can have permanent and devastating effects on the offspring and the family. The risks of infant morbidity and mortality should be accounted for when recommending GWG ranges for optimizing pregnancy outcomes. Based on analysis of nationwide birth and infant death data from 2011 to 2015, this study aims to assess the optimal GWG ranges associated with reduced risks of infant morbidity and mortality across BMI categories.

## Methods

### Study Participants

We used birth cohort linked birth and infant death data sets from the National Center for Health Statistics (NCHS) database, registered in all 50 states in the US and Washington, DC.^18^ We included data sets from 2011, when maternal weight and height were first reported, to the latest available year of 2015. Maternal and infant information on the birth certificate was linked to the infant death certificate, with linkage rates (by year) ranging from 98.8% to 99.4%. This database is deidentified and publicly available and the NCHS assumed responsibility for ethical clearance of data collection and publication. More details of the database can be found from the NCHS.^18^ In the present study, we focused on singleton births and excluded postterm births (gestational age, ≥42 weeks and 0 days [5.6%]), considering that infant deaths in postterm pregnancies are mainly due to utero-placental insufficiency, meconium aspiration, intrauterine infection, or intrapartum and neonatal asphyxia, rather than inappropriate GWG.^19,20^ We also excluded incomplete records (12.8%) missing on GWG or BMI (eFigure in the Supplement). This study followed the Strengthening the Reporting of Observational Studies in Epidemiology (STROBE) reporting guideline for cohort studies.^21^

### Assessment of BMI and GWG

Prepregnancy weight and height were self-reported or based on the mother’s prenatal care records.^22^ Maternal weight at birth was derived from labor and delivery records, admission history, or physical examination.^22^ Body mass index, calculated as prepregnancy weight in kilograms divided by height in meters squared, was categorized into 6 groups according to the World Health Organization (WHO) classification: underweight (<18.5), normal weight (18.5-24.9), overweight (25.0-29.9), obesity class 1 (30.0-34.9), obesity class 2 (35.0-39.9), and obesity class 3 (≥40.0). In the present study, we used GWG equivalent to 40 weeks by standardizing the total GWG (the difference between prepregnancy weight and weight at delivery) with gestational age (eMethods in the Supplement),^11,23,24^ which allowed comparisons of GWG with different lengths of gestation. The GWG equivalent to 40 weeks was then classified into 2.0-kg groups, from weight loss (GWG <0 kg) to weight gain of 30 kg or more, to assess the optimal ranges of GWG.

### Assessment of Adverse Infant Outcomes

We adopted 2 composite outcome measures: (1) significant morbidity of the newborn infant (derived from medical records),^22^ defined as any presence of assisted ventilation, admission to the NICU, surfactant therapy, antibiotic therapy, or seizures; and (2) infant mortality younger than 1 year of age (reported by medical examiners or coroners),^25^ consisting of death in less than 1 hour, 1 to 23 hours, 1 to 6 days, 7 to 27 days, and 28 to 365 days after birth, excluding nonnatural death (accident, suicide, homicide and self-inflicted death, and death from pending or undetermined causes). The definition of each type of morbidity was provided in the specifications of birth certificate.^22^

### Statistical Analysis

Statistical analysis was performed from February 11 to October 14, 2021. Given the effect modification of BMI,^2,5,6,7,8,9^ all statistical analyses were stratified by the 6 BMI categories. We calculated the prevalence of composite and individual outcomes across GWG groups. Adjusted odds ratios (AORs) for composite outcomes were estimated by comparing the odds of each GWG group with the odds of all the rest of the groups,^2^ using logistic regression models adjusted for putative confounders that were associated with GWG (eTable 1 in the Supplement), including maternal age (<25, 25-34, and ≥35 years; categorical), race and ethnicity (as BMI classifications vary by race and ethnicity), educational level, marital status, smoking before or during pregnancy, parity, sex of the infant, place of birth, and type of health insurance.^26,27^ We did not adjust for other pregnancy outcomes that could act as mediators between GWG and infant outcomes, such as maternal complications during pregnancy.^2,28^ We calculated 95% CIs of AORs based on the normal distribution. The optimal GWG range was defined as all GWG groups with significant risk estimates (AOR) less than 1, and GWG groups with nonsignificant AORs less than 1 but between 2 significant AORs less than 1. The overall optimal GWG range was defined as the overlapping GWG ranges for reduced risks of infant morbidity and mortality (eMethods in the Supplement).

We performed 5 sensitivity analyses. First, we reanalyzed the associations in infants born in 2015 to reduce the risk of selection bias, given the lowest rate of missing values in GWG or BMI (5.5%; eFigure in the Supplement). Second, we corrected *P* values to control type I error of multiple testing, using the Benjamini-Hochberg approach that was statistically powerful for large numbers of comparisons.^29^ Third, we reanalyzed the associations in a subset restricted to Hispanic, non-Hispanic Black, and non-Hispanic White races and ethnicities, given that a different BMI classification is used for evaluating body fat percentages in Asian populations.^30^ Fourth, neural tube defects could be associated with low birth weight and insufficient GWG, but were less likely to result from inappropriate GWG^4^; hence, they were excluded to address potential reverse causality. We did not exclude other congenital anomalies that could be associated with inappropriate GWG, such as congenital heart disease.^31^ Fifth, infant morbidity regarding specific disorders was examined by removing NICU admission from the composite variable of morbidity. Missing data of variables used in this study were described in eFigure and eTable 2 in the Supplement. All statistical tests were 2-sided and *P* < .05 denoted statistical significance. All statistical analyses were performed in R, version 3.6.2 (R Group for Statistical Computing).

## Results

Of the 15 759 945 mother-infant dyads (mean [SD] maternal age, 28.1 [5.9] years), 4.0% of women were underweight, 47.0% were normal weight, 25.3% were overweight, 13.2% had class 1 obesity, 6.2% had class 2 obesity, and 4.3% had class 3 obesity (eTable 3 in the Supplement). Women gained a mean (SD) of 14.1 (7.3) kg during pregnancy, and the mean (SD) GWG decreased with increasing BMI categories (underweight, 15.7 [6.4] kg; normal weight, 15.4 [6.2] kg; overweight, 14.2 [7.4] kg; obesity class 1, 12.2 [8.0] kg; obesity class 2, 10.3 [8.4] kg; obesity class 3, 8.2 [9.2] kg; *P* < .001 for trend) (eTable 1 in the Supplement).

eTable 4 in the Supplement shows that 8.8% of the newborns experienced significant morbidity, with the lowest prevalence among infants delivered by normal weight women (8.0%) and the highest among infants delivered by women with class 3 obesity (12.4%); 3.4 per 1000 infants (0.34%) died within 1 year of birth, with the lowest prevalence among infants delivered by normal weight women (0.28%) and the highest among infants delivered by women with class 3 obesity (0.58%).

**Figure 1.**
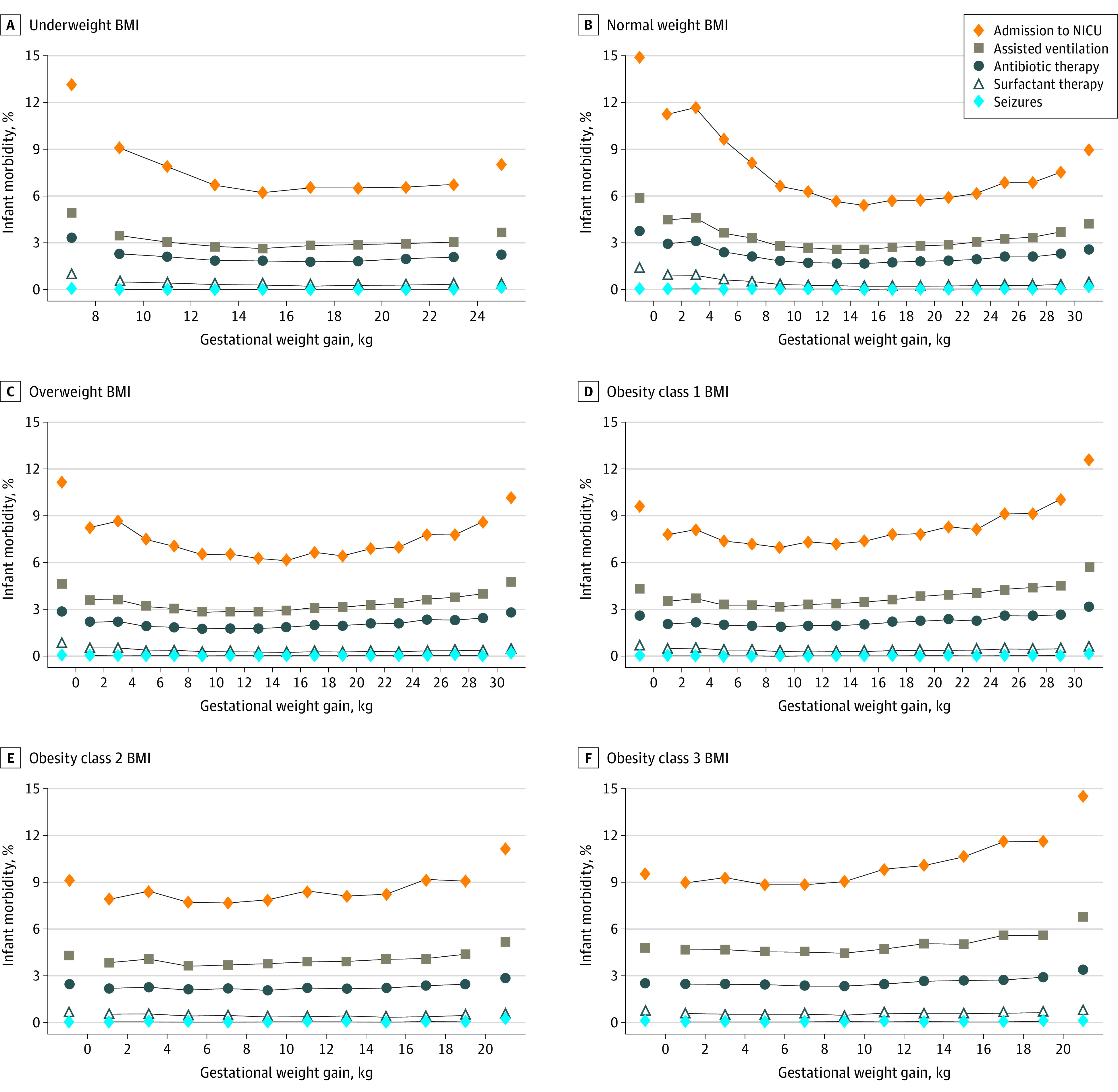
Infant Morbidity Rates by Gestational Weight Gain and Body Mass Index (BMI) A, Underweight BMI (n = 628 929). B, Normal weight BMI (n = 7 410 061). C, Overweight BMI (n = 3 987 800). D, Obesity class 1 BMI (n = 2 075 150). E, Obesity class 2 BMI (n = 980 500). F, Obesity class 3 BMI (n = 677 505). Gestational weight gain was in 2.0-kg groups, and extreme groups were combined into markers on both sides. The numerical results are provided in eTable 5 in the Supplement. NICU indicates neonatal intensive care unit.

**Figure 2.**
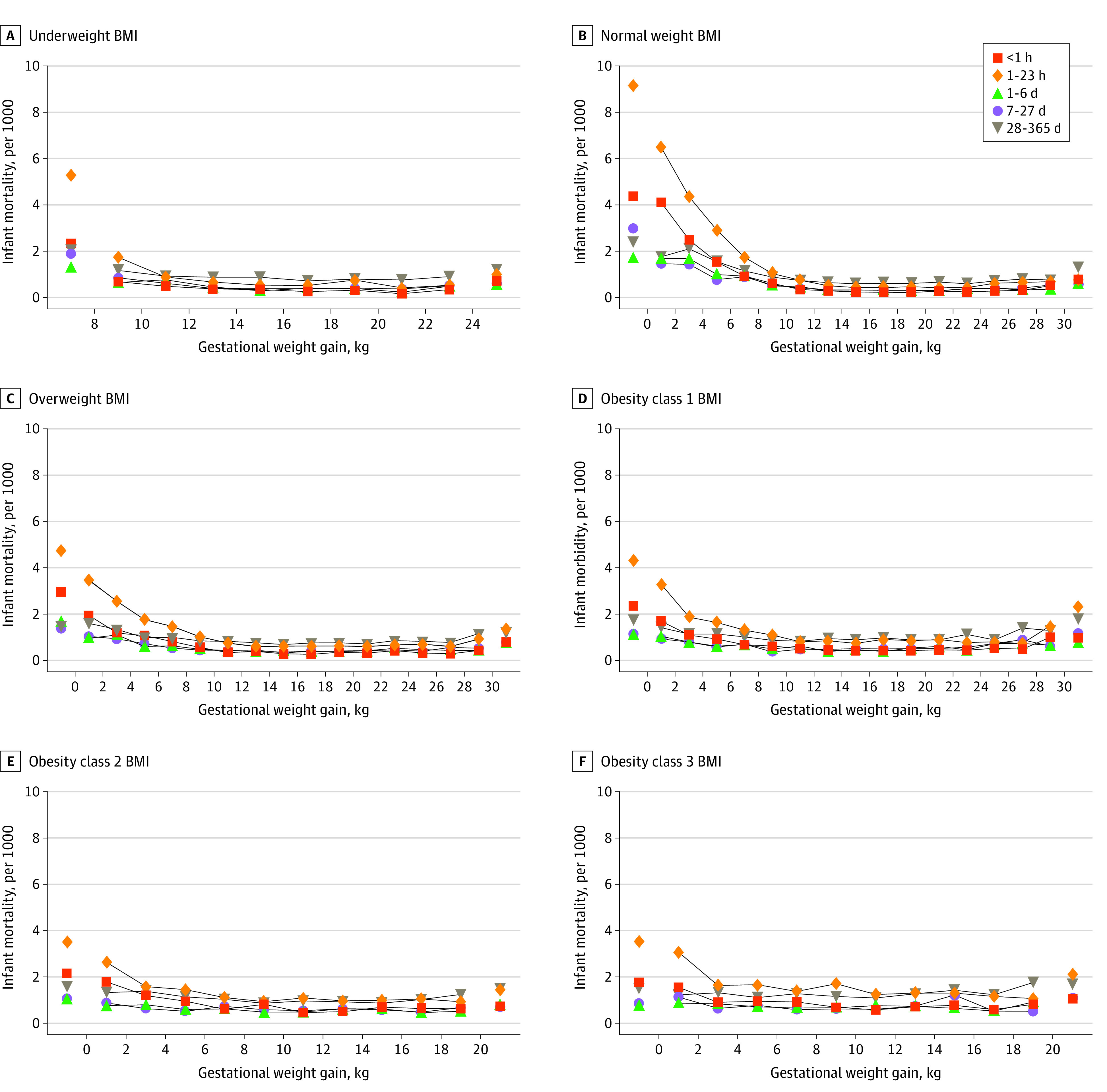
Infant Mortality Rates by Gestational Weight Gain and Body Mass Index (BMI) A, Underweight BMI (n = 628 929). B, Normal weight BMI (n = 7 410 061). C, Overweight BMI (n = 3 987 800). D, Obesity class 1 BMI (n = 2 075 150). E, Obesity class 2 BMI (n = 980 500). F, Obesity class 3 BMI (n = 677 505). Gestational weight gain was in 2.0-kg groups, and extreme groups were combined into markers on both sides. The numerical results are provided in eTable 6 in the Supplement.

The absolute risks of infant morbidity and mortality were lowest in the middle groups of GWG, with U-shaped patterns (Figure 1 and Figure 2) (numerical results in eTables 5 and 6 in the Supplement). Infant morbidity (Figure 1) was most prevalent in the lowest GWG groups among underweight (<8 kg GWG, 14.7%) and normal weight women (<0 kg GWG, 16.6%) and most prevalent in the highest GWG groups among women with obesity (obesity class 1: ≥30 kg GWG, 15.2%; obesity class 2: ≥20 kg GWG, 13.7%; obesity class 3: ≥20 kg GWG, 17.4%) (eTable 5 in the Supplement). Infant mortality (Figure 2) was most prevalent in the lowest GWG groups and remained at low levels in middle and high GWG groups across BMI categories.

**Figure 3.**
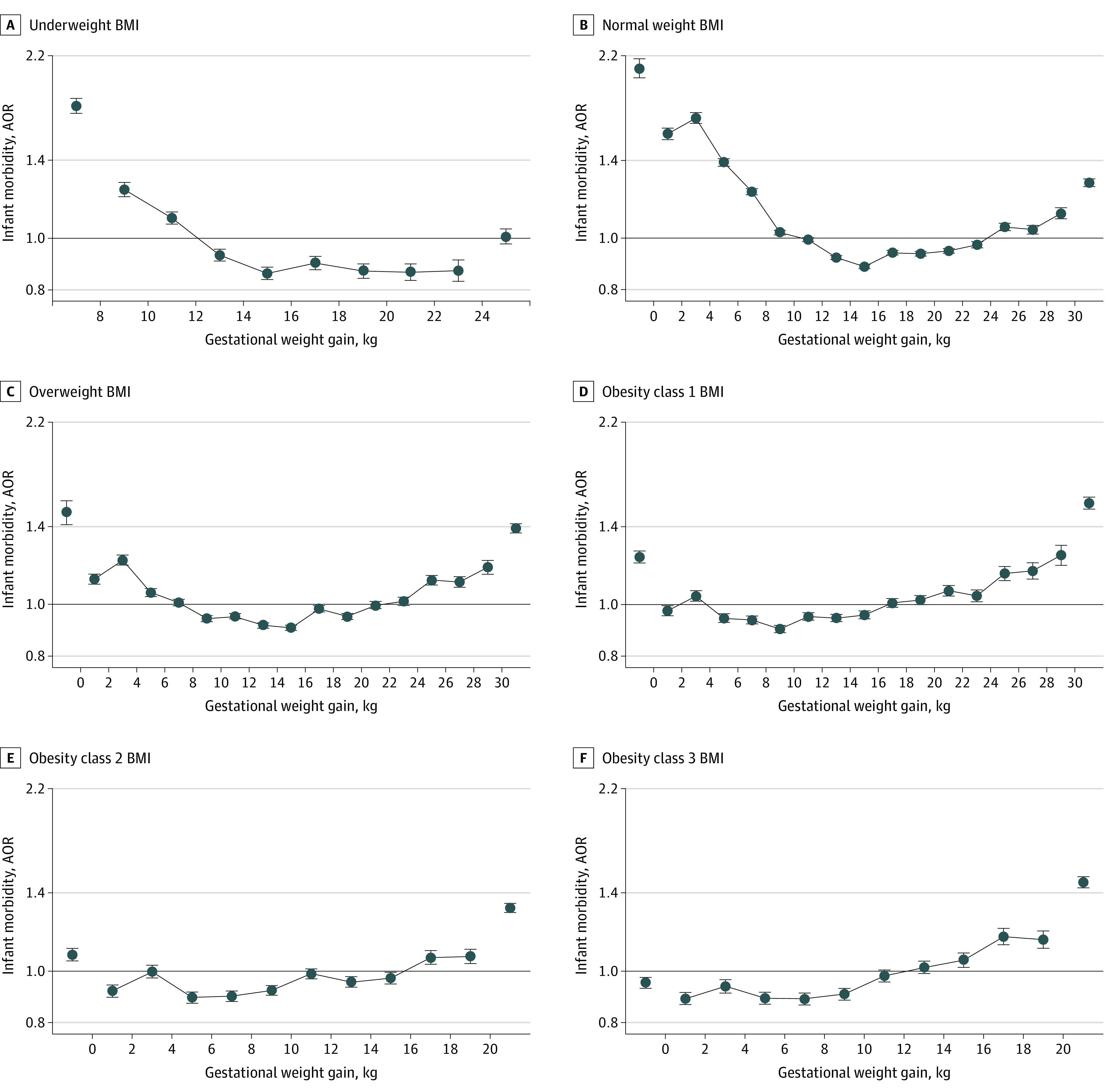
Associations Between Gestational Weight Gain and Infant Morbidity by Body Mass Index (BMI) A, Underweight BMI (n = 628 929). B, Normal weight BMI (n = 7 410 061). C, Overweight BMI (n = 3 987 800). D, Obesity class 1 BMI (n = 2 075 150). E, Obesity class 2 BMI (n = 980 500). F, Obesity class 3 BMI (n = 677 505). Gestational weight gain was in 2.0-kg groups, and extreme groups were combined into markers on both sides. Adjusted odds ratios (AORs) were adjusted for age (<25, 25-34, and ≥35 years; categorical), race and ethnicity, educational level, marital status, smoking before or during pregnancy, parity, sex of infant, place of birth, and type of health insurance. The numerical results are shown in eTable 7 in the Supplement.

**Figure 4.**
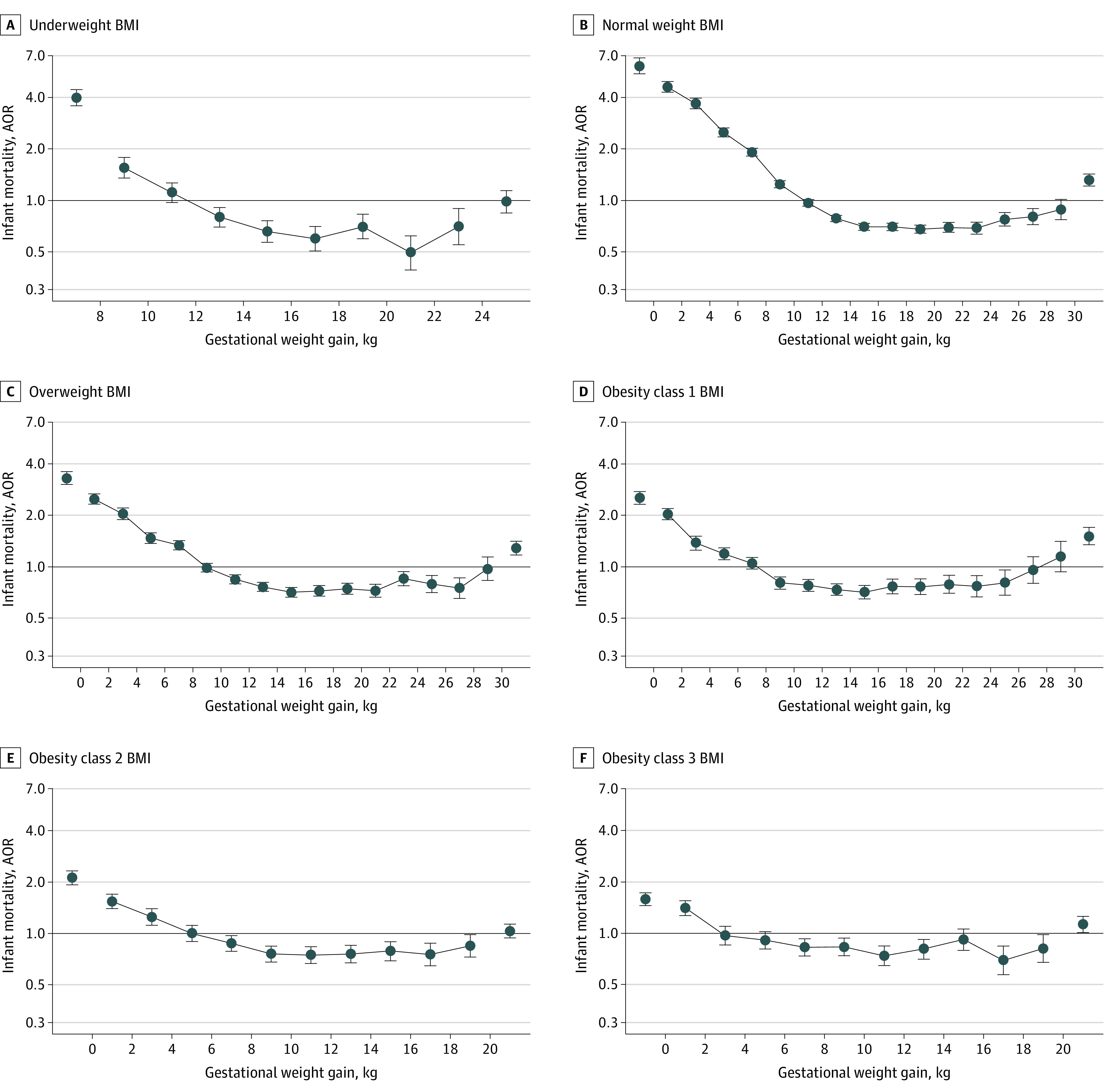
Associations Between Gestational Weight Gain and Infant Mortality by Body Mass Index (BMI) A, Underweight BMI (n = 628 929). B, Normal weight BMI (n = 7 410 061). C, Overweight BMI (n = 3 987 800). D, Obesity class 1 BMI (n = 2 075 150). E, Obesity class 2 BMI (n = 980 500). F, Obesity class 3 BMI (n = 677 505). Gestational weight gain was in 2.0-kg groups, and extreme groups were combined into markers on both sides. Adjusted odds ratios (AORs) were adjusted for age (<25, 25-34, and ≥35 years; categorical), race and ethnicity, educational level, marital status, smoking before or during pregnancy, parity, sex of infant, place of birth, and type of health insurance. The numerical results are shown in eTable 8 in the Supplement.

The associations of GWG with infant morbidity (Figure 3) and mortality (Figure 4) also showed U-shaped patterns. Middle groups of GWG were associated with AORs less than 1, and extremely low and high GWG groups were associated with AORs greater than 1 (numerical results in eTables 7 and 8 in the Supplement). The curvilinear associations interacted with BMI categories. Infant mortality was associated with GWG less than 6.0 kg for women with class 1 obesity (4.0 to <6.0 kg GWG: AOR, 1.19 [95% CI, 1.10-1.29]; 2.0 to <4.0 kg GWG: AOR, 1.37 [95% CI, 1.25-1.51]; 0 to <2.0 kg GWG: AOR, 2.03 [95% CI, 1.88-2.19]; <0 kg GWG: AOR, 2.53 [95% CI, 2.33-2.75]), GWG less than 4.0 kg for women with class 2 obesity (2.0 to <4.0 kg GWG: AOR, 1.25 [95% CI, 1.11-1.39]; 0 to <2.0 kg GWG: AOR, 1.54 [1.40-1.69]; <0 kg GWG: AOR, 2.11 [95% CI, 1.93-2.32]), and GWG less than 2.0 kg for women with class 3 obesity (0 to <2.0 kg GWG: AOR, 1.40 [95% CI, 1.27-1.55]; <0 kg GWG: AOR, 1.58 [95% CI, 1.45-1.72]) (eTable 8 in the Supplement).

**Figure 5.**
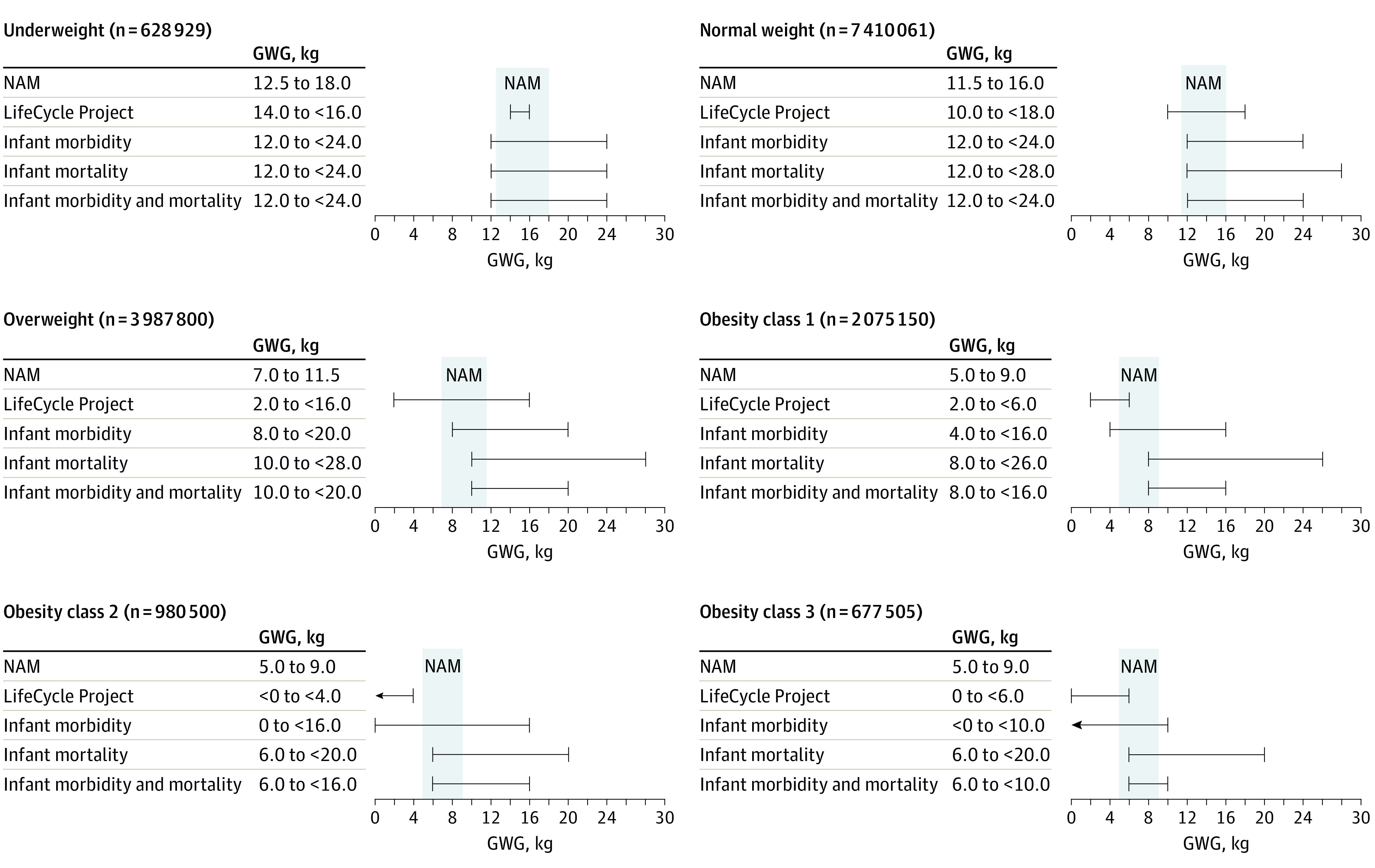
Optimal Gestational Weight Gain (GWG) Ranges From National Academy of Medicine (NAM) Recommendations, LifeCycle Project, and the Present Study GWG, gestational weight gain. The existing NAM recommendations^4^ were based on worldwide studies and evidence by 2009. The LifeCycle Project^2^ analyzed individual data from 25 cohort studies from Europe and North America by 2015. “Infant morbidity” and “infant mortality” denote the optimal ranges of GWG for reduced risks for the respective outcomes, estimated by the present study. “Infant morbidity and mortality” are the overlapping ranges for the 2 composite outcomes of infant morbidity and infant mortality. The LifeCycle Project and the present study used similar statistical methods of regression analyses.

The overall optimal GWG ranges for reduced infant morbidity and mortality were 12.0 to less than 24.0 kg for women with underweight and normal weight BMIs, 10.0 to less than 20.0 kg for women with overweight BMIs, 8.0 to less than 16.0 kg for women with class 1 obesity, 6.0 to less than 16.0 kg for women with class 2 obesity, and 6.0 to less than 10.0 kg for women with class 3 obesity (Figure 5). Comparing NAM recommendations, the LifeCycle Project, and the present study, the results showed that the optimal ranges from the present study were wider, with higher upper bounds. With regard to the lower bounds, our results approximated to NAM recommendations for women with underweight (12.0 vs 12.5 kg) and normal weight (12.0 vs 11.5 kg) BMIs, but for women with overweight BMIs and women with class 1, 2, or 3 obesity, our results (10.0, 8.0, 6.0, and 6.0 kg, respectively) appeared to be higher than those from the LifeCycle Project (2.0, 2.0, <0, and 0 kg, respectively) and NAM recommendations (7.0, 5.0, 5.0, and 5.0 kg, respectively).

In the first sensitivity analysis, the AORs among births in 2015 were comparable to those from the main analysis, while the optimal GWG ranges became slightly narrower, possibly owing to the smaller sample size and fewer significant 95% CIs (eTables 9 and 10 in the Supplement). The other sensitivity analyses showed that the optimal GWG ranges were almost unchanged with corrected *P* values (eTables 11 and 12 in the Supplement), after excluding women of non-Hispanic other races and ethnicities (eTables 13 and 14 in the Supplement), excluding infants with neural tube defects (eTables 15 and 16 in the Supplement), and excluding NICU admission (eTable 17 in the Supplement).

## Discussion

We analyzed data from 2011 to 2015 from a nationwide database and explored optimal GWG ranges for reducing adverse infant outcomes across BMI categories. Associations between GWG and adverse pregnancy outcomes were modified by BMI. It has been reported that the associations between inadequate GWG and infant mortality were stronger among women without obesity,^9^ and our results also showed that the ranges of optimal GWG generally decreased with BMI categories and the severity of obesity. Recommending a single GWG range (5.0-9.0 kg) for women with obesity may be of concern, especially with increasing obesity in pregnant women (increased by 8% from 2011 to 2015^32^). Research based on maternal outcomes and intermediate infant outcomes has suggested lower levels of GWG or even weight loss for women with obesity.^2,5,6,7,8^ However, Bodnar et al^11^ analyzed Pennsylvania birth and infant death data from 2003 to 2011, showing that weight loss and low GWG (*z* score <−1 SD) were associated with increased risks of infant mortality in 219 000 women with class 1 or 2 obesity. We categorized GWG into 2.0-kg groups and found that increased risks of infant mortality were significantly associated with GWG less than 6.0 kg for women with class 1 obesity, less than 4.0 kg for women with class 2 obesity, and less than 2.0 kg for women with class 3 obesity. Our study corroborates the finding by Bodnar et al^11^ that weight maintenance and weight loss during pregnancy could increase adverse infant outcomes even for women with obesity.

Our results added direct evidence to the increased health risk to offspring of inadequate GWG. There are several causal explanations. First, insufficient GWG is associated with being born small for gestational age, preterm birth, and decreased head circumference,^1,2,33^ and in turn linked to higher risks of hypoglycemia, hypocalcemia, immaturity of the respiratory system, serious infections, and cerebral palsy due to hypoxia.^34,35,36,37^ Second, low GWG can reflect a severe negative energy balance and a possible decrease in body mass of the mother, given that approximately 6 to 7 kg in the total GWG is composed of the products of conception (fetus, placenta, and amniotic fluid) and expansion of blood and other extracellular fluid.^38^ Such a negative energy balance could be associated with the viability of the fetus.^39^ Alternatively, low GWG may be a physiological sign of suboptimal health conditions, such as depression and anxiety, prediabetes, and suboptimal fetal and placental development.^34,40^

Excessive GWG has been associated with increased risks of adverse maternal outcomes and infants born large for gestational age for women with an overweight BMI and women with obesity,^2,4^ and our results showed similar associations with infant morbidity and mortality. The mechanisms for adverse outcomes associated with excessive GWG remain understudied, but researchers have explored the pathologic characteristics of pregnancy among obese women. Obesity-related insulin resistance and hyperinsulinemia is associated with placental functions and neonatal hypoglycemia, which can cause severe brain damage if untreated.^41,42^ This could partially explain the increased risks from excessive GWG, considering that neonatal hypoglycemia was also observed among women who gained weight above the recommendations.^17^ A higher inflammatory state in placental and fetal tissue is another potential explanation, which has been reported to be associated with maternal obesity and excessive GWG.^43,44^

The associations of inappropriate GWG with adverse infant outcomes could be different from those with maternal outcomes. Excessive GWG can be associated with larger adverse effects on the mother than the fetus, and is linked to markedly increased risks of pregnancy complications, cesarean delivery, and postpartum weight retention.^2,45,46,47^ Conversely, reducing GWG among women with obesity may trade better maternal outcomes with higher risks for infants. This is not a simple balancing between risks and benefits for the mother and the offspring. For women with obesity without chronic diseases and pregnancy complications related to obesity, minimal GWG that is safe for the offspring is recommended. For women with extreme obesity (BMI ≥40) or those with obesity-related diseases, prenatal care clinicians may adopt individualized GWG recommendations after comprehensive evaluations, and weight maintenance and weight loss should be recommended with great caution. Optimizing nutrient intake and appropriate physical exercise could be a possible way to minimize weight gain and reduce risks for both mothers and infants.

### Strengths and Limitations

This study has some strengths, including directly addressing the ultimate health outcomes of the offspring, including morbidity and mortality; the large sample that allowed for cross-stratification by BMI and GWG; and a relatively short time span of 5 years to avoid effect modification of social transition. In addition, we corrected GWG by gestation length. Given that methods of standardizing GWG by gestation length have not yet been established, most previous studies used total GWG,^2,27,48^ which could overestimate the associations owing to the confounder of gestation length. Some studies adjusted for it in regression analyses,^2,49,50^ which also would be problematic, as preterm and postterm were usually considered as intermediate outcomes in the pathway from risk factors to adverse infant outcomes. Therefore, we used GWG equivalent at 40 weeks to reduce misclassification between appropriate and inappropriate GWG for different gestation lengths, and to facilitate comparisons with the existing recommendations.^23^ In addition, we separately examined infant morbidity and mortality, instead of creating a composite variable of any adverse infant outcomes, given the large disparities in the prevalence. The overall optimal GWG range was defined as the overlapping part to avoid automatic weighting by the prevalence when merging the 2 outcomes.

This study also has some limitations. First, prepregnancy weight and height from birth certificates were reported by mothers.^22^ Studies showed that they were generally representative and consistent with medical records^51,52^ but could be less reliable at the extremes.^53^ Second, the sensitivity and detection rates of some adverse conditions were low,^54^ but the associations were unlikely to be biased considering that sensitivity would not vary with GWG. Third, information on spontaneous abortion and stillbirth was unavailable, which could be associated with underestimation of the effect sizes (AORs) of inappropriate GWG. However, these severe events were associated with more extreme GWG ranges,^55^ and less likely to change the results of optimal GWG ranges. Fourth, various types of morbidity were considered as equally important when merged into the composite variable of “infant morbidity,” although this process automatically weighted each morbidity by its prevalence. Fifth, standardizing GWG to 40 weeks based on a linear pattern could be inaccurate, because the rate of GWG is lower in the first and late third trimesters in an S-shaped pattern. However, this method attenuated misclassification and facilitated comparisons with existing recommendations.^23^ Sixth, the optimal ranges from our results were relatively wide, as we used severe outcomes that were more likely to occur in extreme intrauterine environments. Maternal outcomes should also be accounted for to avoid maternal adverse effects associated with excessive GWG. Seventh, the remaining confounders, such as alcohol drinking, physical activity, and diet, could bias the associations. Further studies are warranted to examine the associations of GWG with maternal and infant outcomes independent of other health risk behaviors.

## Conclusions

This cohort study of more than 15.8 million mother-infant dyads found that the extremes of GWG were associated with increased risks of adverse infant outcomes across BMI categories, suggesting that weight maintenance and weight loss should not be used as routine guidelines, even for women with obesity.

## Notes

Supplement. **eFigure.** Flow Chart of Inclusion and Exclusion Criteria of the Sample in Main Analyses
eMethods.
**eTable 1.** Associations of Background Characteristics and Gestational Weight Gain in 2011–2015 in the US (N=15,759,945) **eTable 2.** Description of Missing Data (N=15,759,945) **eTable 3.** Background Characteristics of the Subjects in 2011-2015 in the US, by Prepregnancy Body Mass Index **eTable 4.** Gestational Weight Gain by Infant Morbidity and Mortality in 2011-2015 in the US, by Prepregnancy Body Mass Index **eTable 5.** Infant Morbidity Rates in 2011–2015 in the US, by Gestational Weight Gain and Prepregnancy Body Mass Index (N=15,759,945) **eTable 6.** Infant Mortality Rates in 2011–2015 in the US, by Gestational Weight Gain and Prepregnancy Body Mass Index (N=15,759,945) **eTable 7.** Associations Between Gestational Weight Gain and Infant Morbidity in 2011–2015 in the US, by Prepregnancy Body Mass Index (N=15,759,945) **eTable 8.** Associations Between Gestational Weight Gain and Infant Mortality in 2011–2015 in the US, by Prepregnancy Body Mass Index (N=15,759,945) **eTable 9.** Associations Between Gestational Weight Gain and Infant Morbidity in 2015 in the US, by Prepregnancy Body Mass Index (N=3,429,315) **eTable 10.** Associations Between Gestational Weight Gain and Infant Mortality in 2015 in the US, by Prepregnancy Body Mass Index (N=3,429,315) **eTable 11.** Corrected *P* Values of the Associations Between Gestational Weight Gain and Infant Morbidity in 2011–2015 in the US, by Prepregnancy Body Mass Index (N=15,759,945) **eTable 12.** Corrected *P* Values of the Associations Between Gestational Weight Gain and Infant Mortality in 2011–2015 in the US, by Prepregnancy Body Mass Index (N=15,759,945) **eTable 13.** Associations Between Gestational Weight Gain and Infant Morbidity in 2011–2015 in the US, Excluding non-Hispanic Other Races (N=14,393,133) **eTable 14.** Associations Between Gestational Weight Gain and Infant Mortality in 2011–2015 in the US, Excluding non-Hispanic Other Races (N=14,393,133) **eTable 15.** Associations Between Gestational Weight Gain and Infant Morbidity in 2011–2015 in the US, Excluding Infants With Neural Tube Defects, by Prepregnancy Body Mass Index (N=15,756,060) **eTable 16.** Associations Between Gestational Weight Gain and Infant Mortality in 2011–2015 in the US, Excluding Infants With Neural Tube Defects, by Prepregnancy Body Mass Index (N=15,756,060) **eTable 17.** Associations Between Gestational Weight Gain and Infant Morbidity (Excluding NICU Admission) in 2011–2015 in the US, by Prepregnancy Body Mass Index (N=15,759,945)
