# Supplementary material for: Association of Gestational Weight Gain With Infant Morbidity and Mortality in the United States
Source: JAMA Netw Open. 2021 Dec 30;4(12):e2141498. doi: 10.1001/jamanetworkopen.2021.41498 (PMC8719246; doi:10.1001/jamanetworkopen.2021.41498)
Supplement: Supplement. — eFigure. Flow Chart of Inclusion and Exclusion Criteria of the Sample in Main Analyses eMethods. eTable 1. Associations of Background Characteristics and Gestational Weight Gain in 2011–2015 in the US (N=15,759,945) eTable 2. Description of Missing Data (N=15,759,945) eTable 3. Background Characteristics of the Subjects in 2011-2015 in the US, by Prepregnancy Body Mass Index eTable 4. Gestational Weight Gain by Infant Morbidity and Mortality in 2011-2015 in the US, by Prepregnancy Body Mass Index eTable 5. Infant Morbidity Rates in 2011–2015 in the US, by Gestational Weight Gain and Prepregnancy Body Mass Index (N=15,759,945) eTable 6. Infant Mortality Rates in 2011–2015 in the US, by Gestational Weight Gain and Prepregnancy Body Mass Index (N=15,759,945) eTable 7. Associations Between Gestational Weight Gain and Infant Morbidity in 2011–2015 in the US, by Prepregnancy Body Mass Index (N=15,759,945) eTable 8. Associations Between Gestational Weight Gain and Infant Mortality in 2011–2015 in the US, by Prepregnancy Body Mass Index (N=15,759,945) eTable 9. Associations Between Gestational Weight Gain and Infant Morbidity in 2015 in the US, by Prepregnancy Body Mass Index (N=3,429,315) eTable 10. Associations Between Gestational Weight Gain and Infant Mortality in 2015 in the US, by Prepregnancy Body Mass Index (N=3,429,315) eTable 11. Corrected P Values of the Associations Between Gestational Weight Gain and Infant Morbidity in 2011–2015 in the US, by Prepregnancy Body Mass Index (N=15,759,945) eTable 12. Corrected P Values of the Associations Between Gestational Weight Gain and Infant Mortality in 2011–2015 in the US, by Prepregnancy Body Mass Index (N=15,759,945) eTable 13. Associations Between Gestational Weight Gain and Infant Morbidity in 2011–2015 in the US, Excluding non-Hispanic Other Races (N=14,393,133) eTable 14. Associations Between Gestational Weight Gain and Infant Mortality in 2011–2015 in the US, Excluding non-Hispanic Other Races (N=14,393,133) eTable [file jamanetwopen-e2141498-s001.pdf]

Supplementary Online Content

Wang L, Zhang X, Chen T, et al. Association of gestational weight gain with infant morbidity and mortality in the United States. *JAMA Netw Open.* 2021;4(12):e2141498. doi:10.1001/jamanetworkopen.2021.41498

eFigure. Flow Chart of Inclusion and Exclusion Criteria of the Sample in Main Analyses
eMethods.
eTable 1. Associations of Background Characteristics and Gestational Weight Gain in 2011–2015 in the US (N=15,759,945)
eTable 2. Description of Missing Data (N=15,759,945)
eTable 3. Background Characteristics of the Subjects in 2011-2015 in the US, by Prepregnancy Body Mass Index
eTable 4. Gestational Weight Gain by Infant Morbidity and Mortality in 2011-2015 in the US, by Prepregnancy Body Mass Index
eTable 5. Infant Morbidity Rates in 2011–2015 in the US, by Gestational Weight Gain and Prepregnancy Body Mass Index (N=15,759,945)
eTable 6. Infant Mortality Rates in 2011–2015 in the US, by Gestational Weight Gain and Prepregnancy Body Mass Index (N=15,759,945)
eTable 7. Associations Between Gestational Weight Gain and Infant Morbidity in 2011–2015 in the US, by Prepregnancy Body Mass Index (N=15,759,945)
eTable 8. Associations Between Gestational Weight Gain and Infant Mortality in 2011–2015 in the US, by Prepregnancy Body Mass Index (N=15,759,945)
eTable 9. Associations Between Gestational Weight Gain and Infant Morbidity in 2015 in the US, by Prepregnancy Body Mass Index (N=3,429,315)
eTable 10. Associations Between Gestational Weight Gain and Infant Mortality in 2015 in the US, by Prepregnancy Body Mass Index (N=3,429,315)
eTable 11. Corrected *P* Values of the Associations Between Gestational Weight Gain and Infant Morbidity in 2011–2015 in the US, by Prepregnancy Body Mass Index (N=15,759,945)
eTable 12. Corrected *P* Values of the Associations Between Gestational Weight Gain and Infant Mortality in 2011–2015 in the US, by Prepregnancy Body Mass Index (N=15,759,945)
eTable 13. Associations Between Gestational Weight Gain and Infant Morbidity in 2011–2015 in the US, Excluding non-Hispanic Other Races (N=14,393,133)
eTable 14. Associations Between Gestational Weight Gain and Infant Mortality in 2011–2015 in the US, Excluding non-Hispanic Other Races (N=14,393,133)
eTable 15. Associations Between Gestational Weight Gain and Infant Morbidity in 2011–2015 in the US, Excluding Infants With Neural Tube Defects, by Prepregnancy Body Mass Index (N=15,756,060)
eTable 16. Associations Between Gestational Weight Gain and Infant Mortality in 2011–2015 in the US, Excluding Infants With Neural Tube Defects, by Prepregnancy Body Mass Index (N=15,756,060)
eTable 17. Associations Between Gestational Weight Gain and Infant Morbidity (Excluding NICU Admission) in 2011–2015 in the US, by Prepregnancy Body Mass Index (N=15,759,945)

This supplementary material has been provided by the authors to give readers additional information about their work.

© 2021 Wang L et al. *JAMA Network Open.*

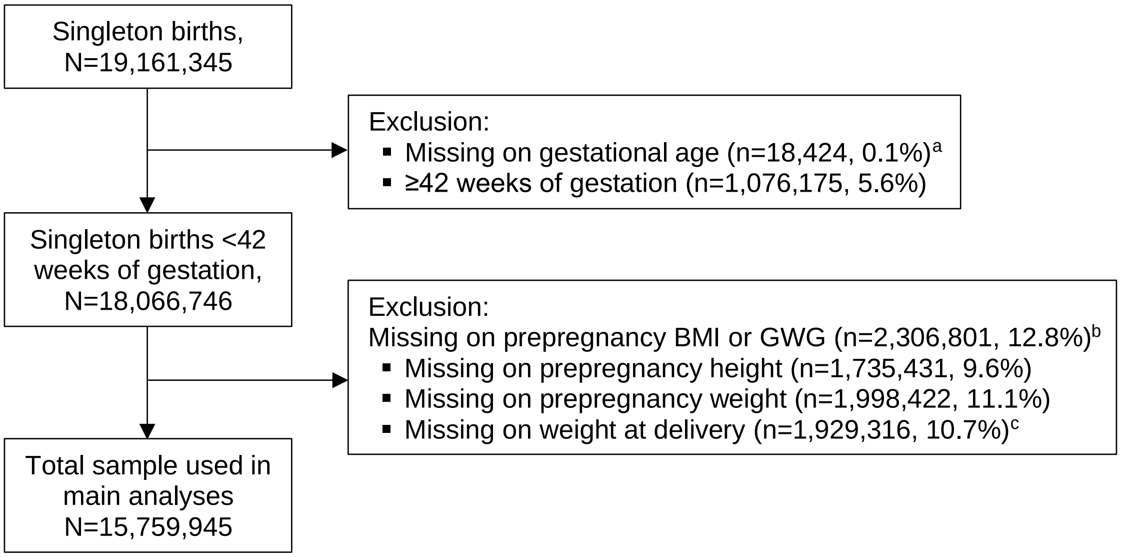

eFigure. Flow Chart of Inclusion and Exclusion Criteria of the Sample in Main Analyses

Abbreviations: BMI, body mass index; GWG, gestational weight gain. [a]Gestational age ranged from 17 to 47 weeks. [b]The number of subjects with missing information on BMI or GWG in each year was 210,743 (5.8%) in 2015, 293,888 (8.1%) in 2014, 518,187 (14.4%) in 2013, 597,456 (16.6%) in 2012, and 686,527 (19.1%) in 2011. Prepregnancy weight and height and weight at delivery were only reported in the latest version of the birth certificate that has been implemented since 2003 and gradually adopted to all states by 2015. The higher missing rates in earlier years could be due to the lower rates of adopting the 2003 birth certificate. [c] 1,263 (0.007%) women with a weight at delivery <100 pound (<45.36 kg) were excluded from the year of 2013, as this extreme category did not present in other years.

© 2021 Wang L et al. *JAMA Network Open.*

eMethods.

The regression analyses were separately performed for each body mass index (BMI) category. The exposure variable was standardized gestational weight gain (GWG) in 2-kg groups. The standardized GWG was calculated by dividing the total GWG (the difference between prepregnancy weight and weight at delivery) by the length of gestation (in weeks) and multiplying by 40. The outcomes were the two composite variables, i.e. infant morbidity and mortality. When assessing the associations, the odds of first GWG group (<0 kg) was compared with the odds of all the rest of the GWG groups (0–<2 kg, 2–<4 kg, 4–<6 kg, etc.) as a whole, yielding the AOR for the first GWG group. Similarly, the AOR of the second GWG group (0–<2 kg) was the ratio of the odds of the second GWG group (0–<2 kg) and the odds of all the rest of the GWG groups (<0 kg, 2–<4 kg, 4–<6 kg, etc.) as a whole.

The optimal GWG range for reduced risks of infant morbidity/mortality was defined as all GWG groups with significant risk estimates less than 1 (AORs <1 and *P* values <.05), and those with non-significant estimates less than 1 (AORs <1 and *P* values ≥.05) but between 2 significant risk estimates less than 1 (both AORs <1 and *P* values <.05). The overall optimal GWG range was defined as the overlapping GWG ranges for reduced risks of infant morbidity and mortality. Extreme GWG groups with insufficient number of events were combined for stable results. For example, underweight women with GWG <8 kg were classified into one group.

© 2021 Wang L et al. *JAMA Network Open.*

eTable 1. Associations of Background Characteristics and Gestational Weight Gain in 2011–2015 in the US (N=15,759,945)

	**Coefficient (95% CI)[a]**
**Age, years**	
<25	-0.04 (-0.05, -0.03)
25-34	Ref
≥35	-0.25 (-0.26, -0.24)
**Race**	
Hispanic	-1.21 (-1.22, -1.20)
Non-Hispanic Black	-0.55 (-0.56, -0.54)
Non-Hispanic White	Ref
Non-Hispanic other[b]	-1.51 (-1.53, -1.50)
**Education**	
High school or below	Ref
Tertiary	0.19 (0.18, 0.20)
**Marital status**	
Unmarried	Ref
Married	-0.53 (-0.54, -0.52)
**Smoking**	
No	Ref
Yes	0.57 (0.56, 0.58)
**Parity**	
Nulliparous	Ref
Multiparous	-1.12 (-1.13, -1.12)
**BMI**	
Underweight	0.28 (0.26, 0.30)
Normal weight	Ref
Overweight	-1.04 (-1.05, -1.03)
Obesity class 1	-2.97 (-2.98, -2.96)
Obesity class 2	-4.95 (-4.97, -4.94)
Obesity class 3	-7.05 (-7.07, -7.04)
P for trend[c]	<.001
**Sex of infant**	
Boy	Ref
Girl	-0.42 (-0.42, -0.41)
**Birth place**	
Hospital/clinic/freestanding birth center	Ref
Home	-0.37 (-0.41, -0.33)
Other	-1.85 (-2.00, -1.70)
**Payment source of delivery**	
Medicaid	Ref
Private insurance	0.39 (0.39, 0.40)
Self-pay	-0.77 (-0.79, -0.75)
Other	0.20 (0.18, 0.22)

[a]Coefficients (95% confidence intervals) were from multivariable linear regression models, adjusted for all variables in this table. [b] Non-Hispanic other races included American Indian/Alaskan Native, Asian, and Native Hawaiian/Other Pacific Islander. [c] *P* for linear trend from underweight to obesity class 3.

© 2021 Wang L et al. *JAMA Network Open.*

**eTable 2.** Description of Missing Data (N=15,759,945)					
	**Information missing**			**Information available**	
	**n**	**%**		**n**	**%**
**Total subjects included in main analyses**				15,759,945	100.0
**Outcomes**					
Significant morbidity of the newborn infant[a]	33,697	0.2		15,726,248	99.8
Infant mortality younger than 1 year of age[b]	0	0.0		15,759,945	100.0
**Covariates**					
Age	0	0.0		15,759,945	100.0
Race	111,032	0.7		15,648,913	99.3
Education level	158,444	1.0		15,601,501	99.0
Marital status	0	0.0		15,759,945	100.0
Smoking before or during pregnancy	525,344	3.3		15,234,601	96.7
Sex of infant	0	0.0		15,759,945	100.0
Place of birth	344	0.0		15,759,601	100.0
Type of health insurance	154,438	1.0		15,605,507	99.0
Parity	130,061	0.8		15,629,884	99.2

[a]Missing on the composite (any morbidity) and individual (assisted ventilation, admission to the neonatal intensive care unit, surfactant therapy, antibiotic therapy and seizures) outcomes of morbidity was identical; [b]Missing on the composite and individual outcomes (<1 hour, 1–23 hours, 1–6 days, 7–27 days, and 28–365 days) of mortality was identical.

© 2021 Wang L et al. *JAMA Network Open.*

eTable 3. Background Characteristics of the Subjects in 2011-2015 in the US, by Prepregnancy Body Mass Index

	**All (N=15,759,945)**			**Underweight (n=628,929)**			**Normal weight (n=7,410,061)**			**Overweight (n=3,987,800)**	
	**%[a]**	**Mean GWG (SD)[b], kg**		**%[a]**	**Mean GWG (SD)[b], kg**		**%[a]**	**Mean GWG (SD)[b], kg**		**%[a]**	**Mean GWG (SD)[b], kg**
**All**	100.0	14.1 (7.3)		4.0	15.7 (6.4)		47.0	15.4 (6.2)		25.3	14.2 (7.4)
**Mean age (SD), years**	28.1 (5.9)	-		26.1 (5.9)	-		28.0 (6.0)	-		28.4 (5.9)	-
<25	29.7	14.4 (7.7)		44.6	16.0 (6.4)		30.3	15.5 (6.8)		28.1	14.4 (7.9)
25-34	55.1	14.0 (7.2)		46.0	15.6 (6.3)		54.9	15.4 (5.9)		55.7	14.3 (7.3)
≥35	15.2	13.6 (7.1)		9.4	15.2 (6.8)		14.7	15.1 (5.8)		16.2	13.6 (7.1)
**Race**											
Hispanic	23.9	13.1 (7.1)		17.8	15.3 (6.4)		21.3	14.5 (6.1)		27.7	13.0 (6.9)
Non-Hispanic Black	14.2	13.5 (8.4)		12.9	15.8 (7.5)		11.0	15.2 (7.2)		15.0	14.1 (8.3)
Non-Hispanic White	53.9	14.7 (7.2)		54.2	16.1 (6.3)		57.7	15.9 (6.0)		50.7	15.1 (7.4)
Non-Hispanic other[c]	8.0	13.6 (6.5)		15.1	14.9 (5.4)		10.0	14.3 (5.6)		6.6	12.7 (7.0)
**Education**											
High school or below	62.2	13.8 (7.8)		68.0	16.0 (6.6)		56.2	15.3 (6.7)		64.1	13.9 (7.7)
Tertiary or above	37.8	14.6 (6.5)		32.0	15.2 (5.8)		43.8	15.5 (5.4)		35.9	14.8 (6.9)
**Marital status**											
Unmarried	40.2	14.1 (8.0)		47.5	16.1 (6.7)		37.0	15.5 (6.9)		40.7	14.2 (8.0)
Married	59.8	14.0 (6.9)		52.5	15.4 (6.0)		63.0	15.3 (5.7)		59.3	14.2 (7.0)
**Smoking before or during pregnancy[d]**											
No	89.0	14.0 (7.2)		83.5	15.5 (6.2)		89.9	15.3 (6.0)		89.5	14.1 (7.3)
Yes	11.0	14.7 (8.6)		16.5	16.8 (6.9)		10.1	16.4 (7.4)		10.5	15.0 (8.6)
**Parity**											
Nulliparous	32.8	15.1 (7.3)		42.8	16.1 (6.2)		36.7	15.9 (6.3)		29.9	15.4 (7.7)
Multiparous	67.2	13.6 (7.3)		57.2	15.5 (6.5)		63.3	15.1 (6.1)		70.1	13.7 (7.3)
**Sex of infant**											
Boy	51.3	14.3 (7.4)		51.2	15.9 (6.4)		51.4	15.6 (6.2)		51.3	14.4 (7.5)
Girl	48.7	13.8 (7.3)		48.8	15.6 (6.3)		48.6	15.2 (6.1)		48.7	14.0 (7.4)
**Birth place**											
Hospital/clinic/ freestanding birth center	99.1	14.1 (7.4)		98.8	15.7 (6.4)		98.8	15.4 (6.2)		99.2	14.2 (7.4)
Home	0.9	13.7 (5.9)		1.1	14.7 (5.1)		1.2	14.6 (5.1)		0.7	13.2 (6.4)
Other	0.1	12.2 (6.9)		0.1	13.6 (5.9)		0.1	13.2 (5.7)		0.1	11.8 (7.3)
**Payment source of delivery**											
Medicaid	43.5	13.7 (7.9)		50.5	16.0 (6.7)		38.6	15.3 (6.8)		44.5	13.7 (7.8)
Private insurance	47.7	14.5 (6.8)		40.0	15.5 (5.9)		52.1	15.6 (5.7)		46.4	14.8 (7.0)
Self-pay	4.1	13.1 (6.8)		5.1	15.3 (6.5)		4.6	14.1 (5.9)		4.1	12.4 (6.9)
Other	4.7	14.0 (7.4)		4.5	15.8 (6.5)		4.6	15.3 (6.3)		5.0	14.1 (7.5)

© 2021 Wang L et al. *JAMA Network Open.*

eTable 3 (Continued). Background characteristics of the subjects in 2011-2015 in the US, by prepregnancy body mass index

	Obesity class 1 (n=2,075,150)			Obesity class 2 (n=980,500)			Obesity class 3 (n=677,505)	
	%[a]	Mean GWG (SD)[b], kg		%[a]	Mean GWG (SD)[b], kg		%[a]	Mean GWG (SD)[b], kg
**All**	13.2	12.2 (8.0)		6.2	10.3 (8.4)		4.3	8.2 (9.2)
**Mean age (SD), years**	28.4 (5.8)	-		28.4 (5.7)	-		28.7 (5.5)	-
<25	28.2	12.6 (8.5)		27.6	10.6 (8.8)		25.4	8.5 (9.5)
25-34	55.5	12.2 (7.9)		56.5	10.3 (8.3)		58.7	8.1 (9.1)
≥35	16.3	11.6 (7.7)		15.9	10.0 (8.2)		15.9	8.3 (9.1)
**Race**								
Hispanic	28.3	11.4 (7.5)		24.9	9.9 (8.0)		21.1	8.2 (8.9)
Non-Hispanic Black	17.9	12.3 (8.8)		20.2	10.5 (9.1)		24.7	8.5 (9.8)
Non-Hispanic White	48.9	12.8 (8.0)		50.9	10.4 (8.3)		50.8	8.1 (9.0)
Non-Hispanic other[c]	4.9	11.4 (7.8)		4.0	10.2 (8.5)		3.4	8.7 (9.6)
**Education**								
High school or below	69.9	12.0 (8.2)		72.1	10.2 (8.6)		74.6	8.2 (9.4)
Tertiary or above	30.1	12.7 (7.6)		27.9	10.6 (7.9)		25.4	8.4 (8.6)
**Marital status**								
Unmarried	44.0	12.5 (8.5)		45.2	10.7 (8.9)		47.4	8.7 (9.7)
Married	56.0	12.1 (7.6)		54.8	10.0 (8.0)		52.6	7.9 (8.8)
**Smoking before or during pregnancy[d]**								
No	88.0	12.2 (7.9)		86.8	10.3 (8.3)		86.3	8.3 (9.1)
Yes	12.0	12.8 (9.2)		13.2	10.5 (9.5)		13.7	8.2 (10.2)
**Parity**								
Nulliparous	26.8	13.7 (8.4)		26.2	11.8 (8.7)		25.9	9.6 (9.4)
Multiparous	73.2	11.7 (7.8)		73.8	9.8 (8.3)		74.1	7.8 (9.1)
**Sex of infant**								
Boy	51.2	12.5 (8.1)		51.2	10.5 (8.4)		51.1	8.5 (9.2)
Girl	48.8	12.0 (8.0)		48.8	10.1 (8.4)		48.9	8.0 (9.2)
**Birth place**								
Hospital/clinic/freestanding birth center	99.4	12.2 (8.0)		99.6	10.3 (8.4)		99.7	8.3 (9.2)
Home	0.5	10.7 (7.0)		0.4	8.5 (7.6)		0.3	6.4 (9.1)
Other	0.0	10.0 (7.5)		0.0	7.5 (8.5)		0.0	5.3 (14.1)
**Payment source of delivery**								
Medicaid	49.3	12.0 (8.4)		51.5	10.3 (8.8)		55.4	8.3 (9.6)
Private insurance	42.5	12.6 (7.7)		41.6	10.4 (8.0)		38.9	8.3 (8.7)
Self-pay	3.5	10.8 (7.5)		2.6	9.5 (8.0)		1.9	7.8 (9.1)
Other	4.6	11.9 (7.9)		4.2	10.1 (8.4)		3.8	8.1 (9.2)

Abbreviations: GWG, gestational weight gain. [a]Column percentages unless otherwise stated. [b]Mean (SD) gestational weight gain (kg) in each category of the covariates. [c]Non-Hispanic other races included American Indian/Alaskan Native, Asian, and Native Hawaiian/Other Pacific Islander. [d]Smoking before or during pregnancy were defined as the average number of cigarettes smoked per day ≥1 in the 3 months prior to pregnancy or during pregnancy.

© 2021 Wang L et al. *JAMA Network Open.*

eTable 4. Gestational Weight Gain by Infant Morbidity and Mortality in 2011-2015 in the US, by Prepregnancy Body Mass Index

	All (N=15,759,945)		Underweight (n=628,929)		Normal weight (n=7,410,061)		Overweight (n=3,987,800)	
	%/‰[a]	Median GWG (IQR)[b], kg	%/‰[a]	Median GWG (IQR)[b], kg	%/‰[a]	Median GWG (IQR)[b], kg	%/‰[a]	Median GWG (IQR)[b], kg
**Morbidity, %**								
Any morbidity[c]	8.8	13.6	9.1	14.4	8.0	14.7	8.7	13.9
		(9.1, 18.6)		(11.0, 18.6)		(10.9, 19.0)		(9.3, 19.1)
Assisted ventilation	3.2	13.8	3.1	14.6	2.9	14.9	3.2	14
		(9.1, 18.6)		(11.1, 18.8)		(11.1, 19.1)		(9.3, 19.1)
Admission to NICU	7.0	13.5	7.4	14.2	6.3	14.5	6.9	13.6
		(8.8, 18.1)		(10.7, 18.6)		(10.7, 18.7)		(9.1, 19.0)
Surfactant therapy	0.3	12.4	0.4	13.2	0.3	13.5	0.3	13
		(7.4, 17.7)		(9.7, 17.6)		(9.1, 18.1)		(7.8, 18.6)
Antibiotic therapy	2.0	13.9	2.1	14.5	1.9	14.9	2.0	14.1
		(9.3, 18.6)		(11.1, 18.6)		(11.1, 19.1)		(9.4, 19.1)
Seizures[d]	0.03	14.1	0.02	16.4	0.03	15	0.03	14.5
		(9.5, 19.1)		(12.6, 21.1)		(11.2, 19.2)		(10.0, 19.9)
**Mortality, ‰**								
All within 1 year	3.4	11.2	3.6	12.7	2.8	12.6	3.3	11.4
		(5.8, 16.7)		(8.7, 17.4)		(7.9, 17.4)		(6.0, 17.3)
In <1 hour	0.5	9.4	0.5	12	0.4	10.9	0.5	9.5
		(4.1, 15.4)		(7.3, 17.2)		(6.0, 16.5)		(4.5, 15.7)
In 1–23 hour	1.0	9.5	1.0	11.2	0.8	11.1	1.0	9.9
		(4.3, 15.6)		(7.1, 16.3)		(6.3, 16.5)		(4.7, 16.2)
In 1–6 days	0.5	11.9	0.5	12.8	0.4	13.2	0.5	12.1
		(7.0, 17.3)		(9.8, 17.6)		(8.7, 17.7)		(7.0, 18.1)
In 7–27 days	0.5	12.1	0.6	12.8	0.4	13.4	0.5	12.3
		(7.0, 17.4)		(9.1, 17.3)		(8.8, 18.1)		(7.0, 18.1)
In 28–365 days	0.9	12.7	1.0	14	0.7	13.9	0.9	12.8
		(7.8, 17.7)		(10.6, 18.6)		(9.7, 18.1)		(8.1, 18.1)

© 2021 Wang L et al. *JAMA Network Open.*

eTable 4 (Continued). Gestational weight gain by infant morbidity and mortality in 2011-2015 in the US, by prepregnancy body mass index

	**Obesity class 1 (n=2,075,150)**			**Obesity class 2 (n=980,500)**			**Obesity class 3 (n=677,505)**	
	**%/‰[a]**	**Median GWG (IQR)[b], kg**		**%/‰[a]**	**Median GWG (IQR)[b], kg**		**%/‰[a]**	**Median GWG (IQR)[b], kg**
**Morbidity, %**								
Any morbidity[c]	9.7	12.1		10.7	10.4		12.4	8.8
		(7.1, 17.7)			(5.2, 16.0)			(3.3, 14.7)
Assisted ventilation	3.6	12.2		4.1	10.2		4.9	8.7
		(7.2, 17.7)			(5.1, 15.9)			(3.1, 14.5)
Admission to NICU	7.8	11.9		8.6	10.4		10.0	9.1
		(7.0, 17.6)			(5.2, 16.1)			(3.3, 14.9)
Surfactant therapy	0.4	11.1		0.5	9.1		0.6	8.1
		(5.7, 17.4)			(3.6, 15.2)			(2.2, 14.3)
Antibiotic therapy	2.1	12.1		2.3	10.2		2.6	8.6
		(7.1, 17.7)			(5.0, 15.9)			(2.9, 14.3)
Seizures[d]	0.03	13		0.05	10.7		0.04	9.3
		(7.7, 18.1)			(6.0, 16.7)			(3.9, 15.2)
**Mortality, ‰**								
All within 1 year	4.1	9.5		4.7	8		5.8	6.7
		(4.0, 15.9)			(2.2, 14.3)			(0.0, 13.1)
In <1 hour	0.7	8.1		0.9	6		1.0	5.1
		(2.5, 14.1)			(0.6, 12.6)			(0.0, 11.9)
In 1–23 hour	1.3	8.2		1.4	6.8		1.9	5
		(2.5, 14.7)			(0.0, 13.6)			(0.0, 11.5)
In 1–6 days	0.6	10.2		0.6	8.6		0.8	7.9
		(5.0, 16.2)			(3.4, 15.0)			(2.3, 13.6)
In 7–27 days	0.6	10.7		0.7	8.7		0.8	7.8
		(5.3, 17.2)			(3.6, 14.7)			(1.3, 14.3)
In 28–365 days	1.0	11.3		1.1	9.1		1.3	8.2
		(5.8, 16.8)			(3.8, 15.8)			(2.7, 14.4)

Abbreviations: GWG, gestational weight gain; NICU, neonatal intensive care unit, IQR, interquartile range. [a]Morbidity rates were in hundreds (%), and mortality rates were in thousands (‰). [b]The distributions of gestational weight gain (kg) in each category of infant morbidity and mortality were non-Normal, and described using median and interquartile range. [c]Any mortality denoted any presence of assisted ventilation, admission to the neonatal intensive care unit, surfactant therapy, antibiotic therapy, or seizures. Multiple types of morbidity could occur in one case. [d]Two decimals were preserved owing to the low prevalence of seizures.

© 2021 Wang L et al. *JAMA Network Open.*

eTable 5. Infant Morbidity Rates in 2011–2015 in the US, by Gestational Weight Gain and Prepregnancy Body Mass Index (N=15,759,945)

Gestational weight gain, kg	Any morbidity		Assisted ventilation		Admission to NICU		Surfactant therapy		Antibiotic therapy		Seizures	
	Case (n)[a]	%[b]	Case (n)[a]	%[b]	Case (n)[a]	%[b]	Case (n)[a]	%[b]	Case (n)[a]	%[b]	Case (n)[a]	%[b]
**Underweight (n=628,929)**												
<8	5,587	14.7	1,862	4.9	4,974	13.1	381	1.0	1,258	3.3	12	0.03
8–<10	5,246	10.5	1,731	3.5	4,528	9.1	249	0.5	1,146	2.3	8	0.02
10–<12	7,526	9.4	2,450	3.1	6,320	7.9	342	0.4	1,696	2.1	15	0.02
12–<14	8,416	8.2	2,831	2.8	6,837	6.7	346	0.3	1,914	1.9	14	0.01
14–<16	7,610	7.8	2,569	2.6	6,050	6.2	289	0.3	1,784	1.8	22	0.02
16–<18	6,281	8.2	2,162	2.8	4,984	6.5	181	0.2	1,361	1.8	19	0.02
18–<20	5,199	8.2	1,830	2.9	4,123	6.5	182	0.3	1,152	1.8	15	0.02
20–<22	3,592	8.4	1,271	3.0	2,816	6.6	124	0.3	857	2.0	12	0.03
22–<24	2,264	8.5	807	3.0	1,791	6.7	91	0.3	553	2.1	5	0.02
≥24	5,048	10.0	1,859	3.7	4,087	8.1	183	0.4	1,165	2.3	28	0.06
**Normal weight (n=7,410,061)**												
<0	3,088	16.6	1,093	5.9	2,773	14.9	267	1.4	702	3.8	13	0.07
0–<2	7,982	12.9	2,771	4.5	6,963	11.3	576	0.9	1,812	2.9	24	0.04
2–<4	9,504	13.4	3,273	4.6	8,306	11.7	658	0.9	2,210	3.1	36	0.05
4–<6	18,146	11.2	5,907	3.6	15,693	9.7	1,005	0.6	3,951	2.4	60	0.04
6–<8	31,771	9.7	10,885	3.3	26,679	8.2	1,723	0.5	6,986	2.1	97	0.03
8–<10	49,468	8.1	16,991	2.8	40,365	6.6	2,048	0.3	11,148	1.8	160	0.03
10–<12	68,150	7.8	23,506	2.7	54,973	6.3	2,504	0.3	15,105	1.7	250	0.03
12–<14	80,998	7.3	28,739	2.6	63,177	5.7	2,674	0.2	18,937	1.7	291	0.03
14–<16	77,301	7.1	28,173	2.6	59,179	5.4	2,362	0.2	18,317	1.7	273	0.03
16–<18	65,290	7.5	23,714	2.7	50,097	5.7	1,927	0.2	15,336	1.8	235	0.03
18–<20	57,273	7.6	21,226	2.8	43,511	5.8	1,669	0.2	13,800	1.8	220	0.03
20–<22	40,463	7.8	14,933	2.9	30,706	5.9	1,214	0.2	9,602	1.8	137	0.03
22–<24	27,152	8.1	10,187	3.1	20,666	6.2	828	0.2	6,477	1.9	106	0.03
24–<26	19,086	8.9	7,008	3.3	14,726	6.9	578	0.3	4,509	2.1	73	0.03
26–<28	12,667	9.0	4,751	3.4	9,707	6.9	373	0.3	2,968	2.1	58	0.04
28–<30	7,793	9.8	2,965	3.7	6,034	7.6	268	0.3	1,838	2.3	26	0.03
≥30	16,820	11.2	6,360	4.2	13,461	9.0	719	0.5	3,811	2.5	53	0.04
**Overweight (n=3,987,800)**												
<0	6,059	12.9	2,174	4.6	5,215	11.1	423	0.9	1,349	2.9	20	0.04
0–<2	10,952	9.9	4,021	3.6	9,163	8.3	585	0.5	2,399	2.2	51	0.05
2–<4	10,486	10.3	3,656	3.6	8,785	8.7	530	0.5	2,272	2.2	20	0.02
4–<6	16,774	9.1	5,883	3.2	13,806	7.5	736	0.4	3,513	1.9	48	0.03
6–<8	24,335	8.6	8,581	3.0	19,824	7.0	1,073	0.4	5,224	1.9	79	0.03
8–<10	32,390	8.1	11,212	2.8	25,954	6.5	1,180	0.3	7,024	1.8	87	0.02

© 2021 Wang L et al. *JAMA Network Open.*

eTable 5 (Continued). Infant morbidity rates in 2011–2015 in the US, by gestational weight gain and prepregnancy body mass index (N=15,759,945)

Gestational weight gain, kg	Any morbidity		Assisted ventilation		Admission to NICU		Surfactant therapy		Antibiotic therapy		Seizures	
	Case (n)[a]	%[b]	Case (n)[a]	%[b]	Case (n)[a]	%[b]	Case (n)[a]	%[b]	Case (n)[a]	%[b]	Case (n)[a]	%[b]
10–<12	37,489	8.2	13,094	2.9	29,934	6.6	1,290	0.3	8,192	1.8	133	0.03
12–<14	39,417	8.0	14,221	2.9	31,002	6.3	1,333	0.3	8,814	1.8	133	0.03
14–<16	35,811	8.0	13,146	2.9	27,602	6.1	1,146	0.3	8,355	1.9	118	0.03
16–<18	30,883	8.6	11,231	3.1	24,054	6.7	997	0.3	7,173	2.0	106	0.03
18–<20	27,930	8.4	10,429	3.1	21,354	6.4	884	0.3	6,557	2.0	103	0.03
20–<22	21,430	8.9	7,968	3.3	16,669	6.9	746	0.3	4,994	2.1	85	0.04
22–<24	15,340	9.1	5,707	3.4	11,777	7.0	462	0.3	3,544	2.1	49	0.03
24–<26	11,570	10.0	4,228	3.7	9,023	7.8	394	0.3	2,711	2.3	52	0.04
26–<28	8,300	10.0	3,132	3.8	6,441	7.8	287	0.3	1,912	2.3	40	0.05
28–<30	5,246	10.9	1,932	4.0	4,167	8.6	185	0.4	1,181	2.4	25	0.05
≥30	13,163	12.6	4,996	4.8	10,670	10.2	604	0.6	2,948	2.8	45	0.04
**Obesity class 1 (n=2,075,150)**												
<0	7,420	11.5	2,780	4.3	6,208	9.6	479	0.7	1,695	2.6	17	0.03
0–<2	10,223	9.5	3,788	3.5	8,375	7.8	533	0.5	2,220	2.1	33	0.03
2–<4	9,446	9.9	3,538	3.7	7,759	8.1	517	0.5	2,079	2.2	33	0.03
4–<6	13,431	9.1	4,862	3.3	10,923	7.4	617	0.4	2,957	2.0	48	0.03
6–<8	17,502	9.0	6,377	3.3	14,027	7.2	803	0.4	3,790	1.9	56	0.03
8–<10	20,300	8.8	7,357	3.2	16,126	7.0	725	0.3	4,396	1.9	58	0.03
10–<12	21,477	9.2	7,771	3.3	17,151	7.3	792	0.3	4,649	2.0	78	0.03
12–<14	20,959	9.2	7,733	3.4	16,480	7.2	738	0.3	4,512	2.0	71	0.03
14–<16	17,807	9.4	6,608	3.5	13,998	7.4	578	0.3	3,901	2.1	69	0.04
16–<18	14,220	9.9	5,243	3.6	11,266	7.8	539	0.4	3,155	2.2	54	0.04
18–<20	12,933	10.0	4,956	3.8	10,142	7.9	492	0.4	2,906	2.3	62	0.05
20–<22	9,608	10.5	3,643	4.0	7,606	8.3	355	0.4	2,156	2.4	42	0.05
22–<24	6,841	10.4	2,652	4.0	5,335	8.1	270	0.4	1,488	2.3	23	0.04
24–<26	5,082	11.4	1,915	4.3	4,061	9.1	209	0.5	1,162	2.6	19	0.04
26–<28	3,894	11.6	1,479	4.4	3,068	9.2	155	0.5	870	2.6	15	0.04
28–<30	2,557	12.5	932	4.5	2,071	10.1	100	0.5	549	2.7	13	0.06
≥30	7,428	15.2	2,792	5.7	6,184	12.6	331	0.7	1,543	3.2	23	0.05
**Obesity class 2 (n=980,500)**												
<0	6,914	11.1	2,675	4.3	5,672	9.1	438	0.7	1,552	2.5	17	0.03
0–<2	7,327	9.9	2,860	3.8	5,877	7.9	403	0.5	1,642	2.2	32	0.04
2–<4	6,778	10.4	2,655	4.1	5,493	8.4	363	0.6	1,475	2.3	28	0.04
4–<6	8,352	9.5	3,169	3.6	6,734	7.7	377	0.4	1,822	2.1	34	0.04
6–<8	9,905	9.6	3,811	3.7	7,891	7.7	482	0.5	2,256	2.2	38	0.04
8–<10	10,917	9.9	4,185	3.8	8,691	7.9	409	0.4	2,281	2.1	52	0.05

© 2021 Wang L et al. *JAMA Network Open.*

eTable 5 (Continued). Infant morbidity rates in 2011–2015 in the US, by gestational weight gain and prepregnancy body mass index (N=15,759,945)

Gestational weight gain, kg	Any morbidity		Assisted ventilation		Admission to NICU		Surfactant therapy		Antibiotic therapy		Seizures	
	Case (n)[a]	%[b]	Case (n)[a]	%[b]	Case (n)[a]	%[b]	Case (n)[a]	%[b]	Case (n)[a]	%[b]	Case (n)[a]	%[b]
10–<12	10,631	10.5	3,940	3.9	8,530	8.4	390	0.4	2,245	2.2	47	0.05
12–<14	9,524	10.3	3,640	3.9	7,543	8.1	394	0.4	2,017	2.2	49	0.05
14–<16	7,666	10.5	2,958	4.1	5,994	8.2	253	0.3	1,617	2.2	28	0.04
16–<18	6,052	11.4	2,182	4.1	4,876	9.2	199	0.4	1,259	2.4	31	0.06
18–<20	5,361	11.5	2,045	4.4	4,234	9.1	215	0.5	1,152	2.5	24	0.05
≥20	14,907	13.7	5,592	5.1	12,214	11.2	634	0.6	3,115	2.9	63	0.06
**Obesity class 3 (n=677,505)**												
<0	9,730	11.8	3,938	4.8	7,872	9.6	581	0.7	2,070	2.5	31	0.04
0–<2	7,462	11.2	3,101	4.7	5,963	9.0	385	0.6	1,642	2.5	26	0.04
2–<4	6,149	11.6	2,489	4.7	4,949	9.3	284	0.5	1,297	2.4	19	0.04
4–<6	7,464	11.2	3,026	4.5	5,904	8.9	354	0.5	1,624	2.4	24	0.04
6–<8	7,832	11.2	3,154	4.5	6,191	8.9	365	0.5	1,628	2.3	29	0.04
8–<10	8,035	11.4	3,130	4.4	6,363	9.0	319	0.5	1,643	2.3	36	0.05
10–<12	7,587	12.3	2,915	4.7	6,101	9.9	363	0.6	1,528	2.5	20	0.03
12–<14	6,707	12.6	2,684	5.1	5,358	10.1	299	0.6	1,407	2.6	29	0.05
14–<16	5,203	13.1	1,997	5.0	4,222	10.6	223	0.6	1,070	2.7	16	0.04
16–<18	4,054	14.3	1,583	5.6	3,284	11.6	169	0.6	769	2.7	10	0.04
18–<20	3,567	14.3	1,393	5.6	2,906	11.6	157	0.6	724	2.9	18	0.07
≥20	10,230	17.4	4,002	6.8	8,553	14.5	478	0.8	2,000	3.4	45	0.08

Abbreviations: NICU, neonatal intensive care unit. [a]Number of cases in each group of gestational weight gain. [b]Prevalence of morbidity in each group of gestational weight gain. [c]Two decimals were preserved owing to the low prevalence of seizures.

© 2021 Wang L et al. *JAMA Network Open.*

eTable 6. Infant Mortality Rates in 2011–2015 in the US, by Gestational Weight Gain and Prepregnancy Body Mass Index (N=15,759,945)

	All within 1 year		In <1 hour		In 1–23 hours		In 1–6 days		In 7–27 days		In 28–365 days	
**Gestational weight gain, kg**	**Case (n)[a]**	**‰[b]**	**Case (n)[a]**	**‰[b]**	**Case (n)[a]**	**‰[b]**	**Case (n)[a]**	**‰[b]**	**Case (n)[a]**	**‰[b]**	**Case (n)[a]**	**‰[b]**
**Underweight (n=628,929)**												
<8	485	12.8	88	2.3	200	5.3	50	1.3	71	1.9	76	2.0
8–<10	255	5.1	35	0.7	87	1.7	33	0.7	42	0.8	58	1.2
10–<12	295	3.7	39	0.5	71	0.9	61	0.8	49	0.6	75	0.9
12–<14	278	2.7	37	0.4	67	0.7	46	0.4	40	0.4	88	0.9
14–<16	234	2.4	33	0.3	51	0.5	28	0.3	38	0.4	84	0.9
16–<18	170	2.2	19	0.2	39	0.5	30	0.4	28	0.4	54	0.7
18–<20	164	2.6	19	0.3	47	0.7	25	0.4	24	0.4	49	0.8
20–<22	83	1.9	7	0.2	18	0.4	10	0.2	16	0.4	32	0.7
22–<24	72	2.7	9	0.3	14	0.5	13	0.5	12	0.5	24	0.9
≥24	207	4.1	38	0.7	51	1.0	29	0.6	30	0.6	59	1.2
**Normal weight (n=7,410,061)**												
<0	386	20.6	82	4.4	171	9.1	32	1.7	56	3.0	45	2.4
0–<2	963	15.5	255	4.1	403	6.5	105	1.7	91	1.5	109	1.8
2–<4	858	12.1	177	2.5	310	4.4	120	1.7	102	1.4	149	2.1
4–<6	1,266	7.8	251	1.5	475	2.9	162	1.0	127	0.8	251	1.5
6–<8	1,834	5.6	300	0.9	569	1.7	298	0.9	289	0.9	378	1.2
8–<10	2,138	3.5	363	0.6	624	1.0	310	0.5	303	0.5	538	0.9
10–<12	2,333	2.7	301	0.3	650	0.7	393	0.4	349	0.4	640	0.7
12–<14	2,363	2.1	312	0.3	580	0.5	372	0.3	376	0.3	723	0.6
14–<16	2,107	1.9	263	0.2	474	0.4	372	0.3	342	0.3	656	0.6
16–<18	1,676	1.9	193	0.2	376	0.4	285	0.3	281	0.3	541	0.6
18–<20	1,445	1.9	168	0.2	350	0.5	240	0.3	226	0.3	461	0.6
20–<22	1,032	2.0	147	0.3	213	0.4	157	0.3	168	0.3	347	0.7
22–<24	682	2.0	79	0.2	145	0.4	135	0.4	125	0.4	198	0.6
24–<26	510	2.4	62	0.3	132	0.6	89	0.4	77	0.4	150	0.7
26–<28	355	2.5	43	0.3	92	0.7	50	0.4	57	0.4	113	0.8
28–<30	225	2.8	41	0.5	55	0.7	27	0.3	42	0.5	60	0.7
≥30	658	4.4	113	0.8	193	1.3	91	0.6	85	0.6	176	1.2
**Overweight (n=3,987,800)**												
<0	575	12.2	138	2.9	221	4.7	79	1.7	65	1.4	72	1.5
0–<2	1,005	9.0	218	2.0	387	3.5	109	1.0	113	1.0	178	1.6
2–<4	724	7.1	121	1.2	261	2.6	110	1.1	97	1.0	135	1.3
4–<6	939	5.1	196	1.1	325	1.8	110	0.6	129	0.7	179	1.0
6–<8	1,248	4.4	229	0.8	413	1.5	172	0.6	152	0.5	282	1.0
8–<10	1,317	3.3	219	0.5	402	1.0	184	0.5	177	0.4	335	0.8

© 2021 Wang L et al. *JAMA Network Open.*

eTable 6 (Continued). Infant mortality rates in 2011–2015 in the US, by gestational weight gain and prepregnancy body mass index (N=15,759,945)

	**All within 1 year**		In <1 hour		**In 1–23 hours**		**In 1–6 days**		**In 7–27 days**		**In 28–365 days**	
**Gestational weight gain, kg**	**Case (n)[a]**	**‰[b]**	**Case (n)[a]**	**‰[b]**	**Case (n)[a]**	**‰[b]**	**Case (n)[a]**	**‰[b]**	**Case (n)[a]**	**‰[b]**	**Case (n)[a]**	**‰[b]**
10–<12	1,272	2.8	162	0.4	338	0.7	198	0.4	193	0.4	381	0.8
12–<14	1,255	2.5	189	0.4	295	0.6	186	0.4	208	0.4	377	0.8
14–<16	1,046	2.3	127	0.3	277	0.6	183	0.4	148	0.3	311	0.7
16–<18	864	2.4	94	0.3	225	0.6	134	0.4	136	0.4	275	0.8
18–<20	833	2.5	114	0.3	211	0.6	120	0.4	135	0.4	253	0.8
20–<22	586	2.4	75	0.3	143	0.6	101	0.4	97	0.4	170	0.7
22–<24	485	2.9	69	0.4	111	0.7	87	0.5	75	0.4	143	0.8
24–<26	312	2.7	37	0.3	81	0.7	55	0.5	44	0.4	95	0.8
26–<28	220	2.7	24	0.3	49	0.6	36	0.4	47	0.6	64	0.8
28–<30	167	3.5	21	0.4	44	0.9	21	0.4	25	0.5	56	1.2
≥30	504	4.8	82	0.8	137	1.3	76	0.7	82	0.8	127	1.2
**Obesity class 1 (n=2,075,150)**												
<0	684	10.6	150	2.3	277	4.3	71	1.1	74	1.1	112	1.7
0–<2	889	8.3	181	1.7	352	3.3	108	1.0	99	0.9	149	1.4
2–<4	531	5.5	102	1.1	177	1.8	73	0.8	73	0.8	106	1.1
4–<6	704	4.7	133	0.9	239	1.6	87	0.6	81	0.5	164	1.1
6–<8	829	4.3	128	0.7	253	1.3	124	0.6	132	0.7	192	1.0
8–<10	764	3.3	136	0.6	245	1.1	110	0.5	79	0.3	194	0.8
10–<12	729	3.1	114	0.5	183	0.8	139	0.6	108	0.5	185	0.8
12–<14	673	2.9	92	0.4	190	0.8	81	0.4	102	0.4	208	0.9
14–<16	549	2.9	74	0.4	136	0.7	85	0.4	90	0.5	164	0.9
16–<18	439	3.0	57	0.4	128	0.9	50	0.3	68	0.5	136	0.9
18–<20	400	3.1	53	0.4	107	0.8	67	0.5	61	0.5	112	0.9
20–<22	293	3.2	39	0.4	80	0.9	53	0.6	44	0.5	77	0.8
22–<24	211	3.2	27	0.4	49	0.7	28	0.4	35	0.5	72	1.1
24–<26	157	3.5	22	0.5	35	0.8	31	0.7	31	0.7	38	0.9
26–<28	137	4.1	15	0.4	23	0.7	24	0.7	29	0.9	46	1.4
28–<30	101	4.9	20	1.0	30	1.5	13	0.6	12	0.6	26	1.3
≥30	336	6.8	46	0.9	112	2.3	37	0.8	56	1.1	85	1.7
**Obesity class 2 (n=980,500)**												
<0	581	9.3	134	2.1	218	3.5	67	1.1	66	1.1	96	1.5
0–<2	550	7.4	133	1.8	198	2.7	56	0.8	64	0.9	99	1.3
2–<4	364	5.6	78	1.2	102	1.6	52	0.8	42	0.6	90	1.4
4–<6	404	4.6	83	0.9	126	1.4	51	0.6	45	0.5	99	1.1
6–<8	425	4.1	63	0.6	114	1.1	67	0.6	74	0.7	107	1.0
8–<10	406	3.7	91	0.8	103	0.9	52	0.5	64	0.6	96	0.9

© 2021 Wang L et al. *JAMA Network Open.*

eTable 6 (Continued). Infant mortality rates in 2011–2015 in the US, by gestational weight gain and prepregnancy body mass index (N=15,759,945)

	**All within 1 year**		**In <1 hour**		**In 1–23 hours**		**In 1–6 days**		**In 7–27 days**		**In 28–365 days**	
**Gestational weight gain, kg**	**Case (n)[a]**	**‰[b]**	**Case (n)[a]**	**‰[b]**	**Case (n)[a]**	**‰[b]**	**Case (n)[a]**	**‰[b]**	**Case (n)[a]**	**‰[b]**	**Case (n)[a]**	**‰[b]**
10–<12	356	3.5	47	0.5	109	1.1	49	0.5	53	0.5	98	1.0
12–<14	338	3.6	48	0.5	91	1.0	57	0.6	55	0.6	87	0.9
14–<16	275	3.8	51	0.7	73	1.0	45	0.6	43	0.6	63	0.9
16–<18	194	3.6	33	0.6	56	1.1	24	0.5	26	0.5	55	1.0
18–<20	185	4.0	29	0.6	43	0.9	24	0.5	32	0.7	57	1.2
≥20	560	5.1	81	0.7	160	1.5	89	0.8	76	0.7	154	1.4
**Obesity class 3 (n=677,505)**												
<0	697	8.4	146	1.8	291	3.5	65	0.8	72	0.9	123	1.5
0–<2	523	7.8	103	1.5	205	3.1	58	0.9	75	1.1	82	1.2
2–<4	285	5.3	48	0.9	87	1.6	45	0.8	35	0.7	70	1.3
4–<6	347	5.2	63	0.9	111	1.7	48	0.7	50	0.7	75	1.1
6–<8	336	4.8	64	0.9	96	1.4	46	0.7	41	0.6	89	1.3
8–<10	341	4.8	49	0.7	120	1.7	47	0.7	44	0.6	81	1.1
10–<12	268	4.3	36	0.6	78	1.3	47	0.8	39	0.6	68	1.1
12–<14	254	4.8	37	0.7	70	1.3	39	0.7	40	0.8	68	1.3
14–<16	213	5.4	31	0.8	52	1.3	25	0.6	49	1.2	56	1.4
16–<18	115	4.1	17	0.6	33	1.2	15	0.5	15	0.5	35	1.2
18–<20	127	5.1	20	0.8	27	1.1	23	0.9	13	0.5	44	1.8
≥20	410	6.9	62	1.1	126	2.1	59	1.0	65	1.1	98	1.7

[a]Number of cases in each group of gestational weight gain. [b]Prevalence (in thousand, ‰) of infant mortality in each group of gestational weight gain.

© 2021 Wang L et al. *JAMA Network Open.*

eTable 7. Associations Between Gestational Weight Gain and Infant Morbidity in 2011–2015 in the US, by Prepregnancy Body Mass Index (N=15,759,945)

Underweight (n=628,929)			Normal weight (n=7,410,061)			Overweight (n=3,987,800)		
GWG, kg	COR (95% CI)	AOR (95% CI)[a]	GWG, kg	COR (95% CI)	AOR (95% CI)[a]	GWG, kg	COR (95% CI)	AOR (95% CI)[a]
			<0	2.28 (2.20, 2.37)	2.09 (2.00, 2.17)	<0	1.56 (1.52, 1.61)	1.49 (1.45, 1.54)
			0–<2	1.71 (1.67, 1.75)	1.57 (1.53, 1.61)	0–<2	1.15 (1.13, 1.17)	1.12 (1.09, 1.14)
			2–<4	1.79 (1.75, 1.83)	1.68 (1.64, 1.72)	2–<4	1.21 (1.19, 1.24)	1.21 (1.19, 1.24)
			4–<6	1.45 (1.43, 1.48)	1.39 (1.37, 1.41)	4–<6	1.05 (1.03, 1.06)	1.05 (1.04, 1.07)
<8	1.82 (1.77, 1.87)	1.77 (1.72, 1.83)	6–<8	1.25 (1.23, 1.26)	1.22 (1.21, 1.24)	6–<8	0.99 (0.97, 1.00)	1.01 (0.99, 1.02)
8–<10	1.20 (1.16, 1.24)	1.23 (1.20, 1.27)	8–<10	1.02 (1.01, 1.03)	1.02 (1.01, 1.04)	8–<10	0.92 (0.91, 0.93)	0.94 (0.93, 0.95)
10–<12	1.05 (1.02, 1.07)	1.09 (1.06, 1.12)	10–<12	0.97 (0.96, 0.97)	0.99 (0.99, 1.00)	10–<12	0.93 (0.92, 0.94)	0.95 (0.94, 0.96)
12–<14	0.89 (0.86, 0.91)	0.93 (0.91, 0.95)	12–<14	0.88 (0.88, 0.89)	0.92 (0.91, 0.93)	12–<14	0.89 (0.88, 0.90)	0.91 (0.90, 0.92)
14–<16	0.83 (0.81, 0.85)	0.86 (0.84, 0.88)	14–<16	0.86 (0.85, 0.86)	0.89 (0.88, 0.89)	14–<16	0.89 (0.88, 0.90)	0.90 (0.89, 0.92)
16–<18	0.89 (0.86, 0.91)	0.90 (0.87, 0.92)	16–<18	0.92 (0.91, 0.93)	0.94 (0.93, 0.95)	16–<18	0.98 (0.96, 0.99)	0.98 (0.97, 0.99)
18–<20	0.89 (0.86, 0.92)	0.87 (0.84, 0.90)	18–<20	0.93 (0.93, 0.94)	0.94 (0.93, 0.94)	18–<20	0.96 (0.94, 0.97)	0.95 (0.94, 0.96)
20–<22	0.91 (0.88, 0.95)	0.86 (0.83, 0.90)	20–<22	0.97 (0.96, 0.98)	0.95 (0.94, 0.96)	20–<22	1.02 (1.00, 1.03)	1.00 (0.98, 1.01)
22–<24	0.93 (0.89, 0.98)	0.87 (0.83, 0.91)	22–<24	1.02 (1.00, 1.03)	0.97 (0.96, 0.98)	22–<24	1.05 (1.03, 1.07)	1.01 (1.00, 1.03)
≥24	1.12 (1.09, 1.16)	1.01 (0.98, 1.04)	24–<26	1.13 (1.11, 1.14)	1.05 (1.03, 1.07)	24–<26	1.17 (1.15, 1.19)	1.11 (1.09, 1.13)
			26–<28	1.14 (1.11, 1.16)	1.04 (1.02, 1.06)	26–<28	1.17 (1.14, 1.20)	1.10 (1.08, 1.13)
			28–<30	1.24 (1.21, 1.27)	1.11 (1.09, 1.14)	28–<30	1.28 (1.24, 1.32)	1.17 (1.14, 1.21)
			≥30	1.46 (1.44, 1.49)	1.27 (1.25, 1.29)	≥30	1.52 (1.50, 1.55)	1.39 (1.36, 1.42)
**Obesity class 1 (n=2,075,150)**			**Obesity class 2 (n=980,500)**			**Obesity class 3 (n=677,505)**		
**GWG, kg**	**COR (95% CI)**	**AOR (95% CI)[a]**	**GWG, kg**	**COR (95% CI)**	**AOR (95% CI)[a]**	**GWG, kg**	**COR (95% CI)**	**AOR (95% CI)[a]**
<0	1.22 (1.19, 1.25)	1.23 (1.20, 1.26)	<0	1.05 (1.02, 1.08)	1.07 (1.05, 1.10)	<0	0.94 (0.92, 0.96)	0.95 (0.93, 0.97)
0–<2	0.98 (0.96, 1.00)	0.97 (0.95, 1.00)	0–<2	0.91 (0.89, 0.93)	0.92 (0.89, 0.94)	0–<2	0.88 (0.86, 0.90)	0.89 (0.87, 0.91)
2–<4	1.02 (1.00, 1.04)	1.04 (1.01, 1.06)	2–<4	0.97 (0.95, 1.00)	1.00 (0.97, 1.02)	2–<4	0.92 (0.89, 0.94)	0.94 (0.91, 0.96)
4–<6	0.92 (0.91, 0.94)	0.94 (0.92, 0.96)	4–<6	0.87 (0.85, 0.89)	0.89 (0.87, 0.91)	4–<6	0.88 (0.85, 0.90)	0.89 (0.87, 0.91)
6–<8	0.91 (0.90, 0.93)	0.94 (0.92, 0.95)	6–<8	0.88 (0.86, 0.90)	0.90 (0.88, 0.92)	6–<8	0.88 (0.86, 0.90)	0.89 (0.86, 0.91)
8–<10	0.88 (0.87, 0.90)	0.90 (0.89, 0.91)	8–<10	0.91 (0.89, 0.93)	0.92 (0.90, 0.94)	8–<10	0.90 (0.88, 0.92)	0.91 (0.88, 0.93)
10–<12	0.94 (0.92, 0.95)	0.95 (0.94, 0.97)	10–<12	0.98 (0.96, 1.00)	0.99 (0.97, 1.01)	10–<12	0.98 (0.96, 1.01)	0.98 (0.96, 1.01)
12–<14	0.93 (0.92, 0.95)	0.94 (0.93, 0.96)	12–<14	0.95 (0.93, 0.97)	0.95 (0.93, 0.98)	12–<14	1.02 (0.99, 1.05)	1.02 (0.99, 1.05)
14–<16	0.96 (0.94, 0.97)	0.96 (0.94, 0.97)	14–<16	0.98 (0.96, 1.01)	0.97 (0.94, 0.99)	14–<16	1.07 (1.03, 1.10)	1.05 (1.02, 1.08)
16–<18	1.02 (1.00, 1.04)	1.01 (0.99, 1.03)	16–<18	1.08 (1.05, 1.11)	1.06 (1.03, 1.09)	16–<18	1.19 (1.15, 1.23)	1.16 (1.12, 1.20)
18–<20	1.04 (1.02, 1.06)	1.02 (1.00, 1.04)	18–<20	1.09 (1.06, 1.12)	1.07 (1.03, 1.10)	18–<20	1.18 (1.14, 1.23)	1.15 (1.10, 1.19)
20–<22	1.09 (1.07, 1.12)	1.06 (1.04, 1.09)	≥20	1.38 (1.36, 1.41)	1.31 (1.29, 1.34)	≥20	1.55 (1.51, 1.58)	1.47 (1.44, 1.51)
22–<24	1.08 (1.06, 1.11)	1.04 (1.01, 1.07)						
24–<26	1.20 (1.17, 1.24)	1.14 (1.11, 1.18)						
26–<28	1.23 (1.19, 1.27)	1.16 (1.12, 1.20)						
28–<30	1.33 (1.27, 1.38)	1.24 (1.19, 1.29)						
≥30	1.69 (1.65, 1.73)	1.55 (1.51, 1.59)						

Abbreviations: GWG, gestational weight gain; COR, crude odds ratio; CI, confidence interval; AOR, adjusted odds ratio. [a]Adjusted for age (<25/25–34/≥35 years, categorical), race, education, marital status, smoking before or during pregnancy, parity, sex of infant, place of birth, and type of health insurance. All *P* values of the curvilinear (quadratic) associations between GWG (continuous) and infant morbidity were <.001. All *P* values of interactions with BMI (linear and quadratic-by-liner interaction terms) were <.001.

© 2021 Wang L et al. *JAMA Network Open.*

eTable 8. Associations Between Gestational Weight Gain and Infant Mortality in 2011–2015 in the US, by Prepregnancy Body Mass Index (N=15,759,945)

**Underweight (n=628,929)**			**Normal weight (n=7,410,061)**			**Overweight (n=3,987,800)**		
**GWG, kg**	**COR (95% CI)**	**AOR (95% CI)[a]**	**GWG, kg**	**COR (95% CI)**	**AOR (95% CI)[a]**	**GWG, kg**	**COR (95% CI)**	**AOR (95% CI)[a]**
			<0	7.59 (6.86, 8.41)	6.10 (5.48, 6.78)	<0	3.81 (3.50, 4.14)	3.31 (3.03, 3.62)
			0–<2	5.81 (5.45, 6.20)	4.60 (4.28, 4.93)	0–<2	2.85 (2.67, 3.04)	2.49 (2.32, 2.67)
			2–<4	4.47 (4.18, 4.79)	3.67 (3.41, 3.95)	2–<4	2.20 (2.04, 2.37)	2.04 (1.88, 2.21)
			4–<6	2.89 (2.73, 3.06)	2.49 (2.34, 2.64)	4–<6	1.56 (1.46, 1.67)	1.47 (1.37, 1.58)
<8	4.33 (3.92, 4.80)	4.00 (3.59, 4.46)	6–<8	2.09 (1.99, 2.19)	1.91 (1.82, 2.01)	6–<8	1.36 (1.28, 1.44)	1.34 (1.26, 1.42)
8–<10	1.49 (1.31, 1.69)	1.55 (1.35, 1.78)	8–<10	1.28 (1.22, 1.34)	1.24 (1.18, 1.30)	8–<10	0.98 (0.93, 1.04)	0.99 (0.93, 1.05)
10–<12	1.03 (0.91, 1.17)	1.11 (0.97, 1.26)	10–<12	0.94 (0.90, 0.98)	0.96 (0.92, 1.01)	10–<12	0.81 (0.77, 0.86)	0.85 (0.80, 0.90)
12–<14	0.73 (0.64, 0.82)	0.80 (0.70, 0.91)	12–<14	0.72 (0.69, 0.75)	0.78 (0.75, 0.82)	12–<14	0.73 (0.69, 0.77)	0.76 (0.72, 0.81)
14–<16	0.63 (0.55, 0.73)	0.66 (0.57, 0.76)	14–<16	0.65 (0.62, 0.68)	0.70 (0.67, 0.73)	14–<16	0.67 (0.62, 0.71)	0.71 (0.66, 0.76)
16–<18	0.59 (0.51, 0.69)	0.60 (0.51, 0.70)	16–<18	0.65 (0.62, 0.69)	0.70 (0.66, 0.74)	16–<18	0.69 (0.65, 0.74)	0.72 (0.67, 0.78)
18–<20	0.70 (0.60, 0.82)	0.70 (0.60, 0.83)	18–<20	0.65 (0.62, 0.69)	0.68 (0.64, 0.72)	18–<20	0.73 (0.68, 0.78)	0.74 (0.69, 0.80)
20–<22	0.52 (0.42, 0.65)	0.49 (0.39, 0.62)	20–<22	0.69 (0.65, 0.73)	0.69 (0.65, 0.74)	20–<22	0.71 (0.65, 0.77)	0.72 (0.66, 0.79)
22–<24	0.75 (0.59, 0.95)	0.70 (0.55, 0.90)	22–<24	0.72 (0.66, 0.77)	0.69 (0.63, 0.75)	22–<24	0.85 (0.78, 0.93)	0.85 (0.78, 0.94)
≥24	1.16 (1.00, 1.34)	0.98 (0.84, 1.14)	24–<26	0.84 (0.77, 0.92)	0.77 (0.71, 0.85)	24–<26	0.80 (0.71, 0.89)	0.79 (0.71, 0.89)
			26–<28	0.89 (0.80, 0.99)	0.80 (0.72, 0.90)	26–<28	0.79 (0.69, 0.90)	0.75 (0.65, 0.86)
			28–<30	1.00 (0.88, 1.14)	0.88 (0.77, 1.01)	28–<30	1.03 (0.89, 1.20)	0.97 (0.83, 1.14)
			≥30	1.58 (1.46, 1.71)	1.31 (1.21, 1.42)	≥30	1.46 (1.33, 1.59)	1.29 (1.17, 1.41)
**Obesity class 1 (n=2,075,150)**			**Obesity class 2 (n=980,500)**			**Obesity class 3 (n=677,505)**		
**GWG, kg**	**COR (95% CI)**	**AOR (95% CI)[a]**	**GWG, kg**	**COR (95% CI)**	**AOR (95% CI)[a]**	**GWG, kg**	**COR (95% CI)**	**AOR (95% CI)[a]**
<0	2.77 (2.56, 2.99)	2.53 (2.33, 2.75)	<0	2.12 (1.94, 2.31)	2.11 (1.93, 2.32)	<0	1.57 (1.44, 1.70)	1.58 (1.45, 1.72)
0–<2	2.16 (2.02, 2.32)	2.03 (1.88, 2.19)	0–<2	1.64 (1.50, 1.79)	1.54 (1.40, 1.69)	0–<2	1.41 (1.29, 1.55)	1.40 (1.27, 1.55)
2–<4	1.39 (1.28, 1.52)	1.37 (1.25, 1.51)	2–<4	1.19 (1.07, 1.33)	1.25 (1.11, 1.39)	2–<4	0.92 (0.81, 1.04)	0.97 (0.85, 1.10)
4–<6	1.19 (1.10, 1.28)	1.19 (1.10, 1.29)	4–<6	0.97 (0.88, 1.08)	1.00 (0.90, 1.11)	4–<6	0.89 (0.79, 0.99)	0.91 (0.81, 1.02)
6–<8	1.05 (0.98, 1.13)	1.05 (0.97, 1.13)	6–<8	0.86 (0.78, 0.95)	0.87 (0.79, 0.97)	6–<8	0.81 (0.73, 0.91)	0.83 (0.74, 0.93)
8–<10	0.79 (0.74, 0.85)	0.80 (0.74, 0.87)	8–<10	0.75 (0.68, 0.83)	0.76 (0.68, 0.84)	8–<10	0.82 (0.73, 0.92)	0.83 (0.74, 0.94)
10–<12	0.74 (0.69, 0.80)	0.78 (0.72, 0.84)	10–<12	0.72 (0.65, 0.80)	0.74 (0.66, 0.83)	10–<12	0.73 (0.64, 0.82)	0.74 (0.65, 0.84)
12–<14	0.70 (0.65, 0.76)	0.74 (0.68, 0.80)	12–<14	0.75 (0.67, 0.84)	0.76 (0.67, 0.85)	12–<14	0.81 (0.72, 0.92)	0.81 (0.71, 0.93)
14–<16	0.69 (0.63, 0.75)	0.71 (0.65, 0.78)	14–<16	0.78 (0.69, 0.89)	0.79 (0.69, 0.89)	14–<16	0.92 (0.80, 1.06)	0.92 (0.80, 1.07)
16–<18	0.73 (0.67, 0.81)	0.77 (0.70, 0.85)	16–<18	0.76 (0.66, 0.88)	0.75 (0.65, 0.87)	16–<18	0.69 (0.57, 0.83)	0.69 (0.57, 0.84)
18–<20	0.75 (0.68, 0.83)	0.76 (0.69, 0.85)	18–<20	0.83 (0.71, 0.96)	0.85 (0.73, 0.98)	18–<20	0.87 (0.73, 1.04)	0.82 (0.68, 0.99)
20–<22	0.78 (0.69, 0.87)	0.79 (0.70, 0.89)	≥20	1.10 (1.00, 1.20)	1.03 (0.94, 1.13)	≥20	1.23 (1.11, 1.36)	1.13 (1.01, 1.26)
22–<24	0.78 (0.68, 0.90)	0.77 (0.67, 0.89)						
24–<26	0.86 (0.74, 1.01)	0.81 (0.68, 0.96)						
26–<28	1.01 (0.85, 1.19)	0.96 (0.80, 1.14)						
28–<30	1.21 (1.00, 1.48)	1.15 (0.94, 1.41)						
≥30	1.72 (1.54, 1.92)	1.51 (1.35, 1.70)						

Abbreviations: GWG, gestational weight gain; COR, crude odds ratio; CI, confidence interval; AOR, adjusted odds ratio. [a]Adjusted for age (<25/25–34/≥35 years, categorical), race, education, marital status, smoking before or during pregnancy, parity, sex of infant, place of birth, and type of health insurance. All *P* values of the curvilinear (quadratic) associations between GWG (continuous) and infant mortality were <.001. All *P* values of interactions with BMI (linear and quadratic-by-liner interaction terms) were <.001.

© 2021 Wang L et al. *JAMA Network Open.*

eTable 9. Associations Between Gestational Weight Gain and Infant Morbidity in 2015 in the US, by Prepregnancy Body Mass Index (N=3,429,315)

**Underweight (n=130,458)**			**Normal weight (n=1,569,895)**			**Overweight (n=878,130)**		
**GWG, kg**	**COR (95% CI)**	**AOR (95% CI)[a]**	**GWG, kg**	**COR (95% CI)**	**AOR (95% CI)[a]**	**GWG, kg**	**COR (95% CI)**	**AOR (95% CI)[a]**
			<0	2.33 (2.15, 2.53)	2.12 (1.95, 2.31)	<0	1.62 (1.53, 1.71)	1.55 (1.46, 1.64)
			0–<2	1.68 (1.60, 1.77)	1.53 (1.45, 1.61)	0–<2	1.15 (1.10, 1.20)	1.12 (1.08, 1.17)
			2–<4	1.83 (1.75, 1.91)	1.72 (1.64, 1.80)	2–<4	1.18 (1.14, 1.24)	1.19 (1.14, 1.24)
			4–<6	1.40 (1.35, 1.44)	1.34 (1.29, 1.38)	4–<6	1.03 (0.99, 1.06)	1.03 (1.00, 1.07)
<8	1.80 (1.69, 1.92)	1.76 (1.65, 1.88)	6–<8	1.22 (1.19, 1.25)	1.20 (1.17, 1.23)	6–<8	1.00 (0.97, 1.03)	1.02 (0.99, 1.05)
8–10	1.24 (1.17, 1.33)	1.27 (1.19, 1.36)	8–<10	1.01 (0.99, 1.03)	1.02 (1.00, 1.04)	8–<10	0.91 (0.88, 0.93)	0.93 (0.91, 0.95)
10–12	1.04 (0.98, 1.10)	1.09 (1.03, 1.15)	10–<12	0.96 (0.95, 0.98)	1.00 (0.98, 1.01)	10–<12	0.94 (0.92, 0.96)	0.96 (0.94, 0.99)
12–14	0.88 (0.83, 0.93)	0.93 (0.88, 0.98)	12–<14	0.87 (0.86, 0.88)	0.90 (0.89, 0.92)	12–<14	0.87 (0.85, 0.89)	0.90 (0.88, 0.92)
14–16	0.82 (0.77, 0.86)	0.85 (0.80, 0.90)	14–<16	0.88 (0.86, 0.89)	0.91 (0.89, 0.93)	14–<16	0.89 (0.87, 0.91)	0.90 (0.88, 0.92)
16–18	0.86 (0.81, 0.91)	0.87 (0.81, 0.92)	16–<18	0.91 (0.89, 0.92)	0.93 (0.91, 0.95)	16–<18	0.96 (0.94, 0.99)	0.97 (0.94, 0.99)
18–20	0.86 (0.80, 0.91)	0.83 (0.78, 0.89)	18–<20	0.93 (0.91, 0.94)	0.93 (0.91, 0.95)	18–<20	0.98 (0.95, 1.00)	0.97 (0.94, 1.00)
20–22	0.96 (0.89, 1.04)	0.90 (0.83, 0.97)	20–<22	0.97 (0.95, 0.99)	0.95 (0.93, 0.97)	20–<22	1.05 (1.02, 1.08)	1.02 (0.99, 1.05)
22–24	0.97 (0.88, 1.07)	0.91 (0.82, 1.00)	22–<24	1.03 (1.00, 1.06)	0.98 (0.95, 1.01)	22–<24	1.06 (1.02, 1.10)	1.02 (0.98, 1.06)
≥24	1.14 (1.07, 1.22)	1.02 (0.95, 1.09)	24–<26	1.15 (1.11, 1.18)	1.07 (1.03, 1.10)	24–<26	1.21 (1.17, 1.26)	1.16 (1.11, 1.21)
			26–<28	1.16 (1.11, 1.21)	1.05 (1.01, 1.10)	26–<28	1.14 (1.09, 1.20)	1.07 (1.02, 1.13)
			28–<30	1.32 (1.26, 1.39)	1.17 (1.11, 1.23)	28–<30	1.26 (1.19, 1.34)	1.15 (1.08, 1.23)
			≥30	1.47 (1.42, 1.52)	1.27 (1.22, 1.31)	≥30	1.52 (1.46, 1.58)	1.39 (1.34, 1.45)
**Obesity class 1 (n=467,461)**			**Obesity class 2 (n=225,383)**			**Obesity class 3 (n=157,988)**		
**GWG, kg**	**COR (95% CI)**	**AOR (95% CI)[a]**	**GWG, kg**	**COR (95% CI)**	**AOR (95% CI)[a]**	**GWG, kg**	**COR (95% CI)**	**AOR (95% CI)[a]**
<0	1.22 (1.15, 1.28)	1.22 (1.16, 1.29)	<0	1.00 (0.95, 1.05)	1.02 (0.97, 1.08)	<0	0.89 (0.85, 0.93)	0.92 (0.88, 0.96)
0–<2	0.99 (0.94, 1.03)	0.98 (0.94, 1.03)	0–<2	0.91 (0.86, 0.96)	0.92 (0.87, 0.97)	0–<2	0.87 (0.82, 0.91)	0.88 (0.83, 0.93)
2–<4	1.04 (0.99, 1.09)	1.06 (1.01, 1.11)	2–<4	0.98 (0.93, 1.03)	1.02 (0.96, 1.07)	2–<4	0.96 (0.91, 1.01)	0.98 (0.93, 1.03)
4–<6	0.93 (0.90, 0.97)	0.95 (0.92, 0.99)	4–<6	0.89 (0.85, 0.93)	0.91 (0.86, 0.95)	4–<6	0.87 (0.83, 0.92)	0.89 (0.85, 0.94)
6–<8	0.91 (0.88, 0.94)	0.93 (0.90, 0.97)	6–<8	0.86 (0.82, 0.90)	0.88 (0.84, 0.92)	6–<8	0.89 (0.85, 0.94)	0.90 (0.86, 0.95)
8–<10	0.85 (0.82, 0.88)	0.87 (0.84, 0.90)	8–<10	0.93 (0.89, 0.97)	0.94 (0.90, 0.98)	8–<10	0.91 (0.87, 0.96)	0.91 (0.86, 0.96)
10–<12	0.95 (0.92, 0.98)	0.96 (0.93, 0.99)	10–<12	1.01 (0.97, 1.06)	1.02 (0.97, 1.06)	10–<12	0.95 (0.91, 1.00)	0.96 (0.91, 1.01)
12–<14	0.91 (0.88, 0.94)	0.92 (0.89, 0.95)	12–<14	0.92 (0.88, 0.96)	0.92 (0.88, 0.96)	12–<14	1.07 (1.02, 1.13)	1.06 (1.01, 1.12)
14–<16	0.96 (0.93, 1.00)	0.96 (0.93, 0.99)	14–<16	0.97 (0.92, 1.02)	0.96 (0.91, 1.01)	14–<16	1.06 (1.00, 1.13)	1.04 (0.98, 1.10)
16–<18	1.01 (0.97, 1.05)	1.00 (0.97, 1.04)	16–<18	1.14 (1.08, 1.21)	1.12 (1.06, 1.19)	16–<18	1.09 (1.02, 1.18)	1.06 (0.98, 1.14)
18–<20	1.07 (1.03, 1.11)	1.05 (1.01, 1.09)	18–<20	1.10 (1.04, 1.17)	1.06 (0.99, 1.12)	18–<20	1.21 (1.12, 1.30)	1.17 (1.09, 1.26)
20–<22	1.11 (1.06, 1.16)	1.08 (1.03, 1.13)	≥20	1.38 (1.33, 1.43)	1.31 (1.25, 1.36)	≥20	1.60 (1.53, 1.67)	1.51 (1.44, 1.59)
22–<24	1.10 (1.04, 1.16)	1.05 (1.00, 1.11)						
24–<26	1.19 (1.12, 1.27)	1.14 (1.07, 1.21)						
26–<28	1.28 (1.19, 1.37)	1.21 (1.13, 1.30)						
28–<30	1.36 (1.25, 1.48)	1.28 (1.17, 1.40)						
≥30	1.70 (1.61, 1.79)	1.56 (1.48, 1.65)						

Abbreviations: GWG, gestational weight gain; COR, crude odds ratio; CI, confidence interval; AOR, adjusted odds ratio. [a]Adjusted for age (<25/25–34/≥35 years, categorical), race, education, marital status, smoking before or during pregnancy, parity, sex of infant, place of birth, and type of health insurance.

© 2021 Wang L et al. *JAMA Network Open.*

eTable 10. Associations Between Gestational Weight Gain and Infant Mortality in 2015 in the US, by Prepregnancy Body Mass Index (N=3,429,315)

**Underweight (n=130,458)**			**Normal weight (n=1,569,895)**			**Overweight (n=878,130)**		
**GWG, kg**	**COR (95% CI)**	**AOR (95% CI)[a]**	**GWG, kg**	**COR (95% CI)**	**AOR (95% CI)[a]**	**GWG, kg**	**COR (95% CI)**	**AOR (95% CI)[a]**
			<0	8.26 (6.7, 10.19)	6.62 (5.34, 8.22)	<0	3.69 (3.07, 4.43)	3.19 (2.64, 3.86)
			0–<2	5.42 (4.69, 6.27)	4.34 (3.73, 5.05)	0–<2	2.92 (2.54, 3.36)	2.50 (2.16, 2.90)
			2–<4	4.56 (3.95, 5.26)	3.59 (3.08, 4.18)	2–<4	2.37 (2.03, 2.76)	2.25 (1.92, 2.63)
			4–<6	2.66 (2.34, 3.02)	2.32 (2.03, 2.64)	4–<6	1.49 (1.29, 1.73)	1.42 (1.22, 1.65)
<8	4.64 (3.74, 5.75)	4.20 (3.35, 5.26)	6–<8	1.95 (1.75, 2.17)	1.79 (1.61, 2.00)	6–<8	1.42 (1.25, 1.60)	1.37 (1.21, 1.55)
8–10	1.64 (1.24, 2.16)	1.76 (1.33, 2.32)	8–<10	1.35 (1.23, 1.48)	1.33 (1.21, 1.47)	8–<10	1.03 (0.91, 1.16)	1.06 (0.94, 1.19)
10–12	0.98 (0.75, 1.29)	1.07 (0.81, 1.41)	10–<12	0.89 (0.81, 0.98)	0.93 (0.84, 1.02)	10–<12	0.83 (0.73, 0.94)	0.86 (0.76, 0.98)
12–14	0.75 (0.57, 0.98)	0.83 (0.63, 1.09)	12–<14	0.68 (0.62, 0.74)	0.73 (0.66, 0.80)	12–<14	0.72 (0.63, 0.81)	0.73 (0.64, 0.83)
14–16	0.52 (0.38, 0.72)	0.55 (0.40, 0.77)	14–<16	0.64 (0.58, 0.71)	0.70 (0.63, 0.78)	14–<16	0.67 (0.59, 0.77)	0.72 (0.62, 0.82)
16–18	0.59 (0.42, 0.84)	0.56 (0.39, 0.81)	16–<18	0.65 (0.59, 0.73)	0.69 (0.61, 0.77)	16–<18	0.66 (0.56, 0.76)	0.70 (0.60, 0.82)
18–20	0.75 (0.54, 1.06)	0.77 (0.54, 1.08)	18–<20	0.67 (0.60, 0.75)	0.67 (0.60, 0.76)	18–<20	0.73 (0.63, 0.85)	0.73 (0.63, 0.86)
20–22	0.52 (0.32, 0.84)	0.45 (0.27, 0.75)	20–<22	0.76 (0.66, 0.86)	0.76 (0.66, 0.86)	20–<22	0.70 (0.58, 0.84)	0.70 (0.58, 0.85)
22–24	0.77 (0.46, 1.29)	0.65 (0.37, 1.13)	22–<24	0.69 (0.58, 0.82)	0.68 (0.57, 0.81)	22–<24	0.75 (0.61, 0.93)	0.73 (0.58, 0.90)
≥24	0.96 (0.69, 1.35)	0.84 (0.59, 1.19)	24–<26	0.90 (0.75, 1.08)	0.83 (0.69, 1.01)	24–<26	0.80 (0.63, 1.02)	0.80 (0.63, 1.02)
			26–<28	0.95 (0.76, 1.19)	0.84 (0.67, 1.06)	26–<28	0.57 (0.41, 0.80)	0.56 (0.40, 0.79)
			28–<30	1.17 (0.89, 1.52)	1.04 (0.80, 1.37)	28–<30	0.85 (0.59, 1.22)	0.81 (0.56, 1.17)
			≥30	1.64 (1.39, 1.94)	1.32 (1.11, 1.57)	≥30	1.58 (1.31, 1.91)	1.41 (1.16, 1.70)
**Obesity class 1 (n=467,461)**			**Obesity class 2 (n=225,383)**			**Obesity class 3 (n=157,988)**		
**GWG, kg**	**COR (95% CI)**	**AOR (95% CI)[a]**	**GWG, kg**	**COR (95% CI)**	**AOR (95% CI)[a]**	**GWG, kg**	**COR (95% CI)**	**AOR (95% CI)[a]**
<0	2.82 (2.39, 3.32)	2.47 (2.07, 2.94)	<0	2.40 (2.03, 2.85)	2.36 (1.98, 2.81)	<0	1.78 (1.51, 2.09)	1.74 (1.47, 2.06)
0–<2	2.19 (1.89, 2.54)	2.06 (1.76, 2.40)	0–<2	1.71 (1.43, 2.05)	1.67 (1.39, 2.02)	0–<2	1.55 (1.28, 1.87)	1.59 (1.31, 1.93)
2–<4	1.31 (1.08, 1.58)	1.29 (1.06, 1.56)	2–<4	1.27 (1.03, 1.56)	1.35 (1.09, 1.67)	2–<4	0.89 (0.69, 1.14)	0.87 (0.67, 1.14)
4–<6	1.21 (1.03, 1.42)	1.23 (1.04, 1.45)	4–<6	0.89 (0.72, 1.10)	0.94 (0.75, 1.16)	4–<6	0.93 (0.74, 1.16)	0.98 (0.78, 1.23)
6–<8	1.21 (1.05, 1.40)	1.23 (1.06, 1.43)	6–<8	0.82 (0.67, 1.01)	0.89 (0.73, 1.10)	6–<8	0.78 (0.62, 0.99)	0.81 (0.64, 1.03)
8–<10	0.78 (0.67, 0.91)	0.78 (0.66, 0.92)	8–<10	0.75 (0.61, 0.92)	0.70 (0.56, 0.88)	8–<10	0.63 (0.48, 0.81)	0.65 (0.50, 0.85)
10–<12	0.68 (0.58, 0.81)	0.70 (0.59, 0.83)	10–<12	0.63 (0.50, 0.80)	0.65 (0.51, 0.82)	10–<12	0.61 (0.46, 0.81)	0.65 (0.49, 0.86)
12–<14	0.61 (0.52, 0.73)	0.66 (0.55, 0.79)	12–<14	0.81 (0.65, 1.01)	0.81 (0.64, 1.01)	12–<14	0.79 (0.60, 1.03)	0.78 (0.59, 1.03)
14–<16	0.73 (0.61, 0.87)	0.77 (0.65, 0.93)	14–<16	0.78 (0.61, 1.01)	0.81 (0.63, 1.04)	14–<16	0.78 (0.57, 1.07)	0.77 (0.56, 1.06)
16–<18	0.75 (0.61, 0.92)	0.78 (0.63, 0.95)	16–<18	0.71 (0.52, 0.96)	0.71 (0.52, 0.97)	16–<18	0.66 (0.44, 0.98)	0.67 (0.45, 1.00)
18–<20	0.75 (0.60, 0.92)	0.73 (0.59, 0.92)	18–<20	0.78 (0.57, 1.06)	0.75 (0.54, 1.03)	18–<20	1.14 (0.82, 1.58)	1.05 (0.74, 1.48)
20–<22	0.78 (0.61, 1.00)	0.79 (0.61, 1.02)	≥20	1.06 (0.88, 1.27)	0.96 (0.79, 1.16)	≥20	1.26 (1.02, 1.55)	1.17 (0.94, 1.45)
22–<24	0.65 (0.47, 0.89)	0.65 (0.47, 0.90)						
24–<26	0.71 (0.49, 1.03)	0.69 (0.48, 1.01)						
26–<28	1.02 (0.71, 1.45)	0.98 (0.68, 1.41)						
28–<30	1.29 (0.86, 1.93)	1.26 (0.84, 1.88)						
≥30	1.97 (1.58, 2.44)	1.73 (1.38, 2.17)						

Abbreviations: GWG, gestational weight gain; COR, crude odds ratio; CI, confidence interval; AOR, adjusted odds ratio. [a]Adjusted for age (<25/25–34/≥35 years, categorical), race, education, marital status, smoking before or during pregnancy, parity, sex of infant, place of birth, and type of health insurance.

© 2021 Wang L et al. *JAMA Network Open.*

eTable 11. Corrected *P* Values of the Associations Between Gestational Weight Gain and Infant Morbidity in 2011–2015 in the US, by Prepregnancy Body Mass Index (N=15,759,945)

Underweight (n=628,929)				Normal weight (n=7,410,061)				Overweight (n=3,987,800)		
**GWG, kg**	**Uncorrected P[a]**	**Corrected P[b]**		**GWG, kg**	**Uncorrected P[a]**	**Corrected P[b]**		**GWG, kg**	**Uncorrected P[a]**	**Corrected P[b]**
				<0	<.001	<.001		<0	<.001	<.001
				0–<2	<.001	<.001		0–<2	<.001	<.001
				2–<4	<.001	<.001		2–<4	<.001	<.001
				4–<6	<.001	<.001		4–<6	<.001	<.001
<8	<.001	<.001		6–<8	<.001	<.001		6–<8	.27	.29
8–<10	<.001	<.001		8–<10	<.001	<.001		8–<10	<.001	<.001
10–<12	<.001	<.001		10–<12	.18	.20		10–<12	<.001	<.001
12–<14	<.001	<.001		12–<14	<.001	<.001		12–<14	<.001	<.001
14–<16	<.001	<.001		14–<16	<.001	<.001		14–<16	<.001	<.001
16–<18	<.001	<.001		16–<18	<.001	<.001		16–<18	.005	.006
18–<20	<.001	<.001		18–<20	<.001	<.001		18–<20	<.001	<.001
20–<22	<.001	<.001		20–<22	<.001	<.001		20–<22	.85	.86
22–<24	<.001	<.001		22–<24	<.001	<.001		22–<24	.10	.11
≥24	.61	.62		24–<26	<.001	<.001		24–<26	<.001	<.001
				26–<28	<.001	<.001		26–<28	<.001	<.001
				28–<30	<.001	<.001		28–<30	<.001	<.001
				≥30	<.001	<.001		≥30	<.001	<.001
**Obesity class 1 (n=2,075,150)**				**Obesity class 2 (n=980,500)**				**Obesity class 3 (n=677,505)**		
**GWG, kg**	**Uncorrected P[a]**	**Corrected P[b]**		**GWG, kg**	**Uncorrected P[a]**	**Corrected P[b]**		**GWG, kg**	**Uncorrected P[a]**	**Corrected P[b]**
<0	<.001	<.001		<0	<.001	<.001		<0	<.001	<.001
0–<2	.02	.02		0–<2	<.001	<.001		0–<2	<.001	<.001
2–<4	.001	.002		2–<4	.86	.86		2–<4	<.001	<.001
4–<6	<.001	<.001		4–<6	<.001	<.001		4–<6	<.001	<.001
6–<8	<.001	<.001		6–<8	<.001	<.001		6–<8	<.001	<.001
8–<10	<.001	<.001		8–<10	<.001	<.001		8–<10	<.001	<.001
10–<12	<.001	<.001		10–<12	.29	.30		10–<12	.15	.16
12–<14	<.001	<.001		12–<14	<.001	<.001		12–<14	.24	.25
14–<16	<.001	<.001		14–<16	.02	.02		14–<16	.003	.003
16–<18	.44	.46		16–<18	<.001	<.001		16–<18	<.001	<.001
18–<20	.05	.06		18–<20	<.001	<.001		18–<20	<.001	<.001
20–<22	<.001	<.001		≥20	<.001	<.001		≥20	<.001	<.001
22–<24	.005	.006			<.001	<.001			<.001	<.001
24–<26	<.001	<.001			<.001	<.001			<.001	<.001
26–<28	<.001	<.001			.86	.86			<.001	<.001
28–<30	<.001	<.001			<.001	<.001			<.001	<.001
≥30	<.001	<.001			<.001	<.001			<.001	<.001

Abbreviations: GWG, gestational weight gain. [a] Uncorrected *P* values of associations adjusted for age (<25/25–34/≥35 years, categorical), race, education, marital status, smoking before or during pregnancy, parity, sex of infant, place of birth, and type of health insurance. [b] Corrected *P* values of associations adjusted for age (<25/25–34/≥35 years, categorical), race, education, marital status, smoking before or during pregnancy, parity, sex of infant, place of birth, and type of health insurance, using the Benjamini-Hochberg approach for all comparisons.

© 2021 Wang L et al. *JAMA Network Open.*

eTable 12. Corrected *P* Values of the Associations Between Gestational Weight Gain and Infant Mortality in 2011–2015 in the US, by Prepregnancy Body Mass Index (N=15,759,945)

Underweight (n=628,929)			Normal weight (n=7,410,061)			Overweight (n=3,987,800)		
**GWG, kg**	**Uncorrected P[a]**	**Corrected P[b]**	**GWG, kg**	**Uncorrected P[a]**	**Corrected P[b]**	**GWG, kg**	**Uncorrected P[a]**	**Corrected P[b]**
			<0	<.001	<.001	<0	<.001	<.001
			0–<2	<.001	<.001	0–<2	<.001	<.001
			2–<4	<.001	<.001	2–<4	<.001	<.001
			4–<6	<.001	<.001	4–<6	<.001	<.001
<8	<.001	<.001	6–<8	<.001	<.001	6–<8	<.001	<.001
8–<10	<.001	<.001	8–<10	<.001	<.001	8–<10	.68	.70
10–<12	.12	.14	10–<12	.10	.12	10–<12	<.001	<.001
12–<14	<.001	<.001	12–<14	<.001	<.001	12–<14	<.001	<.001
14–<16	<.001	<.001	14–<16	<.001	<.001	14–<16	<.001	<.001
16–<18	<.001	<.001	16–<18	<.001	<.001	16–<18	<.001	<.001
18–<20	<.001	<.001	18–<20	<.001	<.001	18–<20	<.001	<.001
20–<22	<.001	<.001	20–<22	<.001	<.001	20–<22	<.001	<.001
22–<24	.004	.006	22–<24	<.001	<.001	22–<24	.001	.002
≥24	.79	.79	24–<26	<.001	<.001	24–<26	<.001	<.001
			26–<28	<.001	<.001	26–<28	<.001	<.001
			28–<30	.08	.09	28–<30	.75	.77
			≥30	<.001	<.001	≥30	<.001	<.001
**Obesity class 1 (n=2,075,150)**			**Obesity class 2 (n=980,500)**			**Obesity class 3 (n=677,505)**		
**GWG, kg**	**Uncorrected P[a]**	**Corrected P[b]**	**GWG, kg**	**Uncorrected P[a]**	**Corrected P[b]**	**GWG, kg**	**Uncorrected P[a]**	**Corrected P[b]**
<0	<.001	<.001	<0	<.001	<.001	<0	<.001	<.001
0–<2	<.001	<.001	0–<2	<.001	<.001	0–<2	<.001	<.001
2–<4	<.001	<.001	2–<4	<.001	<.001	2–<4	.61	.65
4–<6	<.001	<.001	4–<6	1.00	1.00	4–<6	.11	.13
6–<8	.22	.24	6–<8	.01	.01	6–<8	.002	.002
8–<10	<.001	<.001	8–<10	<.001	<.001	8–<10	.002	.003
10–<12	<.001	<.001	10–<12	<.001	<.001	10–<12	<.001	<.001
12–<14	<.001	<.001	12–<14	<.001	<.001	12–<14	.002	.003
14–<16	<.001	<.001	14–<16	<.001	<.001	14–<16	.27	.29
16–<18	<.001	<.001	16–<18	<.001	<.001	16–<18	<.001	<.001
18–<20	<.001	<.001	18–<20	.03	.04	18–<20	.03	.04
20–<22	<.001	<.001	≥20	.51	.55	≥20	.03	.03
22–<24	<.001	<.001		<.001	<.001		<.001	<.001
24–<26	.01	.02		<.001	<.001		<.001	<.001
26–<28	.63	.67		<.001	<.001		.61	.65
28–<30	.19	.21		1.00	1.00		.11	.13
≥30	<.001	<.001		.01	.01		.002	.002

Abbreviations: GWG, gestational weight gain. [a] Uncorrected *P* values of associations adjusted for age (<25/25–34/≥35 years, categorical), race, education, marital status, smoking before or during pregnancy, parity, sex of infant, place of birth, and type of health insurance. [b] Corrected *P* values of associations adjusted for age (<25/25–34/≥35 years, categorical), race, education, marital status, smoking before or during pregnancy, parity, sex of infant, place of birth, and type of health insurance, using the Benjamini-Hochberg approach for all comparisons.

© 2021 Wang L et al. *JAMA Network Open.*

eTable 13. Associations Between Gestational Weight Gain and Infant Morbidity in 2011–2015 in the US, Excluding non-Hispanic Other Races (N=14,393,133)

**Underweight (n=529,821)**				**Normal weight (n=6,615,897)**				**Overweight (n=3,699,733)**		
**GWG, kg**	**COR (95% CI)**	**AOR (95% CI)[a]**		**GWG, kg**	**COR (95% CI)**	**AOR (95% CI)[a]**		**GWG, kg**	**COR (95% CI)**	**AOR (95% CI)[a]**
				<0	2.33 (2.24, 2.43)	2.11 (2.02, 2.20)		<0	1.59 (1.54, 1.63)	1.52 (1.47, 1.56)
				0–<2	1.75 (1.71, 1.80)	1.60 (1.56, 1.64)		0–<2	1.17 (1.14, 1.19)	1.13 (1.11, 1.16)
				2–<4	1.83 (1.78, 1.87)	1.70 (1.66, 1.75)		2–<4	1.22 (1.19, 1.24)	1.21 (1.19, 1.24)
				4–<6	1.48 (1.46, 1.51)	1.40 (1.38, 1.43)		4–<6	1.05 (1.03, 1.07)	1.06 (1.04, 1.08)
<8	1.85 (1.79, 1.91)	1.85 (1.79, 1.91)		6–<8	1.26 (1.25, 1.28)	1.24 (1.22, 1.25)		6–<8	0.99 (0.98, 1.00)	1.01 (1.00, 1.03)
8–<10	1.23 (1.19, 1.27)	1.23 (1.19, 1.27)		8–<10	1.03 (1.02, 1.04)	1.03 (1.02, 1.04)		8–<10	0.91 (0.90, 0.93)	0.94 (0.93, 0.95)
10–<12	1.06 (1.03, 1.09)	1.06 (1.03, 1.09)		10–<12	0.97 (0.96, 0.98)	1.00 (0.99, 1.01)		10–<12	0.92 (0.91, 0.93)	0.95 (0.94, 0.96)
12–<14	0.89 (0.87, 0.91)	0.89 (0.87, 0.91)		12–<14	0.89 (0.88, 0.89)	0.92 (0.91, 0.93)		12–<14	0.89 (0.88, 0.90)	0.91 (0.90, 0.92)
14–<16	0.83 (0.81, 0.85)	0.83 (0.81, 0.85)		14–<16	0.85 (0.85, 0.86)	0.88 (0.88, 0.89)		14–<16	0.89 (0.88, 0.90)	0.90 (0.89, 0.91)
16–<18	0.88 (0.86, 0.91)	0.88 (0.86, 0.91)		16–<18	0.91 (0.91, 0.92)	0.94 (0.93, 0.95)		16–<18	0.98 (0.96, 0.99)	0.98 (0.97, 1.00)
18–<20	0.88 (0.85, 0.91)	0.88 (0.85, 0.91)		18–<20	0.92 (0.92, 0.93)	0.93 (0.92, 0.94)		18–<20	0.96 (0.94, 0.97)	0.95 (0.94, 0.96)
20–<22	0.89 (0.86, 0.92)	0.89 (0.86, 0.92)		20–<22	0.96 (0.95, 0.97)	0.94 (0.93, 0.95)		20–<22	1.02 (1.00, 1.03)	1.00 (0.98, 1.01)
22–<24	0.91 (0.87, 0.96)	0.91 (0.87, 0.96)		22–<24	1.01 (0.99, 1.02)	0.97 (0.95, 0.98)		22–<24	1.05 (1.03, 1.07)	1.01 (0.99, 1.03)
≥24	1.08 (1.05, 1.12)	1.08 (1.05, 1.12)		24–<26	1.11 (1.09, 1.13)	1.04 (1.02, 1.06)		24–<26	1.16 (1.14, 1.19)	1.11 (1.09, 1.13)
				26–<28	1.13 (1.10, 1.15)	1.03 (1.01, 1.05)		26–<28	1.17 (1.14, 1.19)	1.10 (1.07, 1.13)
				28–<30	1.24 (1.21, 1.27)	1.11 (1.08, 1.14)		28–<30	1.28 (1.24, 1.32)	1.17 (1.14, 1.21)
				≥30	1.45 (1.43, 1.48)	1.27 (1.25, 1.29)		≥30	1.53 (1.50, 1.56)	1.39 (1.37, 1.42)
**Obesity class 1 (n=1,960,617)**				**Obesity class 2 (n=935,823)**				**Obesity class 3 (n=651,242)**		
**GWG, kg**	**COR (95% CI)**	**AOR (95% CI)[a]**		**GWG, kg**	**COR (95% CI)**	**AOR (95% CI)[a]**		**GWG, kg**	**COR (95% CI)**	**AOR (95% CI)[a]**
<0	1.21 (1.18, 1.24)	1.22 (1.19, 1.25)		<0	1.05 (1.02, 1.07)	1.07 (1.04, 1.10)		<0	0.94 (0.92, 0.96)	0.95 (0.93, 0.97)
0–<2	0.98 (0.96, 1.00)	0.98 (0.95, 1.00)		0–<2	0.92 (0.89, 0.94)	0.92 (0.90, 0.95)		0–<2	0.88 (0.86, 0.90)	0.89 (0.87, 0.92)
2–<4	1.02 (1.00, 1.05)	1.04 (1.01, 1.06)		2–<4	0.97 (0.95, 1.00)	1.00 (0.97, 1.03)		2–<4	0.92 (0.89, 0.94)	0.94 (0.91, 0.96)
4–<6	0.93 (0.91, 0.94)	0.95 (0.93, 0.97)		4–<6	0.87 (0.85, 0.89)	0.89 (0.87, 0.91)		4–<6	0.88 (0.85, 0.90)	0.89 (0.87, 0.92)
6–<8	0.91 (0.89, 0.92)	0.94 (0.92, 0.95)		6–<8	0.88 (0.86, 0.90)	0.89 (0.87, 0.92)		6–<8	0.87 (0.85, 0.90)	0.88 (0.86, 0.91)
8–<10	0.88 (0.87, 0.90)	0.90 (0.89, 0.91)		8–<10	0.91 (0.89, 0.93)	0.92 (0.90, 0.94)		8–<10	0.90 (0.88, 0.92)	0.91 (0.88, 0.93)
10–<12	0.93 (0.92, 0.95)	0.95 (0.94, 0.97)		10–<12	0.98 (0.96, 1.00)	0.99 (0.97, 1.01)		10–<12	0.98 (0.96, 1.01)	0.98 (0.96, 1.01)
12–<14	0.93 (0.92, 0.95)	0.94 (0.93, 0.96)		12–<14	0.95 (0.93, 0.98)	0.96 (0.93, 0.98)		12–<14	1.02 (0.99, 1.05)	1.01 (0.99, 1.04)
14–<16	0.96 (0.94, 0.97)	0.95 (0.94, 0.97)		14–<16	0.98 (0.96, 1.01)	0.97 (0.94, 0.99)		14–<16	1.07 (1.04, 1.11)	1.05 (1.02, 1.09)
16–<18	1.02 (1.00, 1.04)	1.00 (0.99, 1.02)		16–<18	1.08 (1.05, 1.11)	1.06 (1.03, 1.09)		16–<18	1.20 (1.15, 1.24)	1.17 (1.13, 1.21)
18–<20	1.04 (1.02, 1.06)	1.02 (1.00, 1.04)		18–<20	1.09 (1.05, 1.12)	1.06 (1.03, 1.09)		18–<20	1.18 (1.14, 1.22)	1.14 (1.10, 1.19)
20–<22	1.09 (1.07, 1.12)	1.06 (1.03, 1.08)		≥20	1.39 (1.36, 1.41)	1.32 (1.29, 1.34)		≥20	1.54 (1.51, 1.58)	1.47 (1.43, 1.50)
22–<24	1.08 (1.06, 1.11)	1.04 (1.01, 1.07)								
24–<26	1.21 (1.17, 1.25)	1.15 (1.11, 1.19)								
26–<28	1.24 (1.19, 1.28)	1.16 (1.12, 1.20)								
28–<30	1.33 (1.27, 1.38)	1.23 (1.18, 1.29)								
≥30	1.69 (1.65, 1.74)	1.56 (1.51, 1.60)								

Abbreviations: GWG, gestational weight gain; COR, crude odds ratio; CI, confidence interval; AOR, adjusted odds ratio. [a]Adjusted for age (<25/25–34/≥35 years, categorical), race, education, marital status, smoking before or during pregnancy, parity, sex of infant, place of birth, and type of health insurance.

© 2021 Wang L et al. *JAMA Network Open.*

eTable 14. Associations Between Gestational Weight Gain and Infant Mortality in 2011–2015 in the US, Excluding non-Hispanic Other Races (N=14,393,133)

**Underweight (n=529,821)**				**Normal weight (n=6,615,897)**				**Overweight (n=3,699,733)**		
**GWG, kg**	**COR (95% CI)**	**AOR (95% CI)[a]**		**GWG, kg**	**COR (95% CI)**	**AOR (95% CI)[a]**		**GWG, kg**	**COR (95% CI)**	**AOR (95% CI)[a]**
				<0	7.47 (6.71, 8.31)	6.03 (5.40, 6.74)		<0	3.92 (3.60, 4.28)	3.37 (3.07, 3.69)
				0–<2	5.84 (5.45, 6.25)	4.52 (4.20, 4.87)		0–<2	2.87 (2.68, 3.07)	2.51 (2.34, 2.70)
				2–<4	4.48 (4.17, 4.82)	3.67 (3.39, 3.96)		2–<4	2.23 (2.07, 2.42)	2.06 (1.89, 2.23)
				4–<6	2.94 (2.77, 3.12)	2.50 (2.35, 2.66)		4–<6	1.60 (1.50, 1.72)	1.50 (1.40, 1.62)
<8	4.22 (3.80, 4.70)	3.93 (3.51, 4.40)		6–<8	2.10 (2.00, 2.21)	1.90 (1.81, 2.01)		6–<8	1.35 (1.27, 1.44)	1.34 (1.26, 1.43)
8–<10	1.55 (1.35, 1.77)	1.58 (1.37, 1.82)		8–<10	1.30 (1.24, 1.36)	1.25 (1.19, 1.31)		8–<10	0.98 (0.92, 1.04)	0.98 (0.92, 1.04)
10–<12	1.03 (0.90, 1.17)	1.09 (0.95, 1.25)		10–<12	0.95 (0.91, 1.00)	0.97 (0.92, 1.01)		10–<12	0.81 (0.77, 0.86)	0.85 (0.79, 0.90)
12–<14	0.77 (0.68, 0.88)	0.84 (0.73, 0.96)		12–<14	0.73 (0.70, 0.76)	0.78 (0.75, 0.82)		12–<14	0.73 (0.69, 0.78)	0.76 (0.72, 0.81)
14–<16	0.65 (0.57, 0.75)	0.67 (0.57, 0.77)		14–<16	0.65 (0.62, 0.68)	0.70 (0.67, 0.74)		14–<16	0.66 (0.62, 0.71)	0.70 (0.66, 0.75)
16–<18	0.60 (0.51, 0.71)	0.60 (0.51, 0.71)		16–<18	0.66 (0.63, 0.70)	0.71 (0.67, 0.75)		16–<18	0.69 (0.65, 0.75)	0.72 (0.67, 0.78)
18–<20	0.67 (0.57, 0.80)	0.69 (0.58, 0.82)		18–<20	0.65 (0.62, 0.69)	0.68 (0.64, 0.72)		18–<20	0.72 (0.67, 0.78)	0.74 (0.69, 0.80)
20–<22	0.54 (0.43, 0.67)	0.51 (0.40, 0.65)		20–<22	0.69 (0.65, 0.74)	0.70 (0.65, 0.75)		20–<22	0.70 (0.65, 0.77)	0.72 (0.65, 0.78)
22–<24	0.71 (0.55, 0.91)	0.67 (0.52, 0.87)		22–<24	0.71 (0.65, 0.76)	0.69 (0.63, 0.74)		22–<24	0.85 (0.77, 0.93)	0.86 (0.78, 0.94)
≥24	1.07 (0.92, 1.24)	0.94 (0.80, 1.10)		24–<26	0.84 (0.77, 0.92)	0.78 (0.71, 0.86)		24–<26	0.81 (0.72, 0.90)	0.80 (0.71, 0.90)
				26–<28	0.86 (0.77, 0.96)	0.80 (0.72, 0.90)		26–<28	0.78 (0.68, 0.90)	0.75 (0.65, 0.86)
				28–<30	0.99 (0.87, 1.13)	0.89 (0.78, 1.03)		28–<30	1.05 (0.90, 1.22)	0.99 (0.85, 1.17)
				≥30	1.53 (1.41, 1.66)	1.31 (1.20, 1.42)		≥30	1.49 (1.36, 1.63)	1.33 (1.21, 1.46)
**Obesity class 1 (n=1,960,617)**				**Obesity class 2 (n=935,823)**				**Obesity class 3 (n=651,242)**		
**GWG, kg**	**COR (95% CI)**	**AOR (95% CI)[a]**		**GWG, kg**	**COR (95% CI)**	**AOR (95% CI)[a]**		**GWG, kg**	**COR (95% CI)**	**AOR (95% CI)[a]**
<0	2.71 (2.50, 2.94)	2.46 (2.26, 2.69)		<0	2.10 (1.92, 2.30)	2.09 (1.91, 2.30)		<0	1.54 (1.41, 1.67)	1.58 (1.44, 1.72)
0–<2	2.18 (2.03, 2.35)	2.06 (1.91, 2.23)		0–<2	1.61 (1.46, 1.76)	1.52 (1.38, 1.68)		0–<2	1.41 (1.28, 1.54)	1.40 (1.27, 1.55)
2–<4	1.38 (1.26, 1.51)	1.35 (1.23, 1.49)		2–<4	1.20 (1.08, 1.34)	1.26 (1.13, 1.41)		2–<4	0.91 (0.81, 1.03)	0.97 (0.85, 1.10)
4–<6	1.17 (1.08, 1.27)	1.18 (1.09, 1.28)		4–<6	0.98 (0.88, 1.08)	1.01 (0.91, 1.13)		4–<6	0.88 (0.78, 0.98)	0.90 (0.80, 1.01)
6–<8	1.04 (0.97, 1.12)	1.04 (0.96, 1.13)		6–<8	0.86 (0.78, 0.96)	0.87 (0.79, 0.97)		6–<8	0.83 (0.74, 0.92)	0.84 (0.74, 0.94)
8–<10	0.78 (0.72, 0.84)	0.79 (0.73, 0.86)		8–<10	0.75 (0.68, 0.84)	0.77 (0.69, 0.86)		8–<10	0.85 (0.76, 0.95)	0.85 (0.76, 0.96)
10–<12	0.75 (0.70, 0.82)	0.79 (0.73, 0.86)		10–<12	0.73 (0.65, 0.81)	0.75 (0.67, 0.84)		10–<12	0.71 (0.62, 0.80)	0.72 (0.63, 0.82)
12–<14	0.71 (0.66, 0.77)	0.75 (0.69, 0.82)		12–<14	0.74 (0.66, 0.83)	0.75 (0.67, 0.85)		12–<14	0.82 (0.72, 0.93)	0.81 (0.71, 0.93)
14–<16	0.71 (0.65, 0.77)	0.72 (0.66, 0.79)		14–<16	0.79 (0.70, 0.89)	0.79 (0.69, 0.89)		14–<16	0.92 (0.80, 1.06)	0.91 (0.78, 1.05)
16–<18	0.74 (0.67, 0.81)	0.77 (0.69, 0.85)		16–<18	0.76 (0.66, 0.88)	0.75 (0.64, 0.87)		16–<18	0.69 (0.57, 0.83)	0.69 (0.57, 0.84)
18–<20	0.75 (0.68, 0.83)	0.77 (0.69, 0.86)		18–<20	0.82 (0.71, 0.95)	0.83 (0.71, 0.97)		18–<20	0.89 (0.74, 1.07)	0.82 (0.68, 1.00)
20–<22	0.79 (0.70, 0.89)	0.79 (0.70, 0.89)		≥20	1.10 (1.01, 1.21)	1.03 (0.93, 1.13)		≥20	1.24 (1.12, 1.38)	1.14 (1.02, 1.27)
22–<24	0.80 (0.69, 0.92)	0.78 (0.68, 0.90)								
24–<26	0.86 (0.73, 1.02)	0.82 (0.69, 0.97)								
26–<28	0.99 (0.84, 1.18)	0.94 (0.79, 1.13)								
28–<30	1.20 (0.98, 1.47)	1.12 (0.91, 1.39)								
≥30	1.75 (1.57, 1.96)	1.54 (1.37, 1.73)								

Abbreviations: GWG, gestational weight gain; COR, crude odds ratio; CI, confidence interval; AOR, adjusted odds ratio. [a]Adjusted for age (<25/25–34/≥35 years, categorical), race, education, marital status, smoking before or during pregnancy, parity, sex of infant, place of birth, and type of health insurance.

© 2021 Wang L et al. *JAMA Network Open.*

eTable 15. Associations Between Gestational Weight Gain and Infant Morbidity in 2011–2015 in the US, Excluding Infants With Neural Tube Defects, by Prepregnancy Body Mass Index (N=15,756,060)

**Underweight (n=628,788)**			**Normal weight (n=7,408,418)**			**Overweight (n=3,986,817)**		
**GWG, kg**	**COR (95% CI)**	**AOR (95% CI)[a]**	**GWG, kg**	**COR (95% CI)**	**AOR (95% CI)[a]**	**GWG, kg**	**COR (95% CI)**	**AOR (95% CI)[a]**
			<0	2.29 (2.20, 2.38)	2.09 (2.00, 2.17)	<0	1.56 (1.52, 1.61)	1.49 (1.45, 1.54)
			0–<2	1.71 (1.67, 1.75)	1.57 (1.53, 1.61)	0–<2	1.15 (1.13, 1.17)	1.12 (1.09, 1.14)
			2–<4	1.79 (1.75, 1.83)	1.68 (1.64, 1.72)	2–<4	1.21 (1.19, 1.24)	1.21 (1.18, 1.24)
			4–<6	1.45 (1.43, 1.48)	1.39 (1.37, 1.41)	4–<6	1.05 (1.03, 1.06)	1.05 (1.03, 1.07)
<8	1.82 (1.76, 1.87)	1.77 (1.72, 1.83)	6–<8	1.25 (1.23, 1.26)	1.22 (1.21, 1.24)	6–<8	0.99 (0.97, 1.00)	1.01 (0.99, 1.02)
8–<10	1.20 (1.16, 1.24)	1.23 (1.20, 1.27)	8–<10	1.02 (1.01, 1.03)	1.03 (1.01, 1.04)	8–<10	0.92 (0.91, 0.93)	0.94 (0.93, 0.95)
10–<12	1.05 (1.02, 1.07)	1.09 (1.06, 1.12)	10–<12	0.97 (0.96, 0.97)	0.99 (0.99, 1.00)	10–<12	0.93 (0.92, 0.94)	0.95 (0.94, 0.96)
12–<14	0.89 (0.86, 0.91)	0.93 (0.91, 0.95)	12–<14	0.88 (0.88, 0.89)	0.92 (0.91, 0.93)	12–<14	0.89 (0.88, 0.90)	0.91 (0.90, 0.92)
14–<16	0.83 (0.81, 0.85)	0.86 (0.84, 0.88)	14–<16	0.86 (0.85, 0.86)	0.89 (0.88, 0.89)	14–<16	0.89 (0.88, 0.90)	0.90 (0.89, 0.92)
16–<18	0.89 (0.86, 0.91)	0.90 (0.87, 0.92)	16–<18	0.92 (0.91, 0.93)	0.94 (0.93, 0.95)	16–<18	0.98 (0.96, 0.99)	0.98 (0.97, 0.99)
18–<20	0.89 (0.86, 0.92)	0.87 (0.84, 0.90)	18–<20	0.93 (0.93, 0.94)	0.94 (0.93, 0.94)	18–<20	0.96 (0.94, 0.97)	0.95 (0.94, 0.96)
20–<22	0.91 (0.88, 0.95)	0.86 (0.83, 0.90)	20–<22	0.97 (0.96, 0.98)	0.95 (0.94, 0.96)	20–<22	1.02 (1.00, 1.03)	1.00 (0.98, 1.01)
22–<24	0.93 (0.89, 0.98)	0.87 (0.83, 0.91)	22–<24	1.02 (1.00, 1.03)	0.97 (0.96, 0.98)	22–<24	1.05 (1.03, 1.07)	1.01 (1.00, 1.03)
≥24	1.12 (1.09, 1.16)	1.01 (0.98, 1.04)	24–<26	1.13 (1.11, 1.14)	1.05 (1.03, 1.07)	24–<26	1.17 (1.15, 1.19)	1.11 (1.09, 1.14)
			26–<28	1.13 (1.11, 1.16)	1.04 (1.02, 1.06)	26–<28	1.17 (1.14, 1.20)	1.10 (1.08, 1.13)
			28–<30	1.24 (1.22, 1.27)	1.11 (1.09, 1.14)	28–<30	1.28 (1.24, 1.32)	1.17 (1.14, 1.21)
			≥30	1.46 (1.44, 1.49)	1.27 (1.25, 1.29)	≥30	1.52 (1.50, 1.55)	1.39 (1.36, 1.42)
**Obesity class 1 (n=2,074,563)**			**Obesity class 2 (n=980,215)**			**Obesity class 3 (n=677,259)**		
**GWG, kg**	**COR (95% CI)**	**AOR (95% CI)[a]**	**GWG, kg**	**COR (95% CI)**	**AOR (95% CI)[a]**	**GWG, kg**	**COR (95% CI)**	**AOR (95% CI)[a]**
<0	1.22 (1.19, 1.25)	1.23 (1.20, 1.26)	<0	1.05 (1.02, 1.08)	1.07 (1.04, 1.10)	<0	0.94 (0.92, 0.96)	0.95 (0.93, 0.97)
0–<2	0.98 (0.96, 1.00)	0.97 (0.95, 1.00)	0–<2	0.91 (0.89, 0.93)	0.92 (0.89, 0.94)	0–<2	0.88 (0.86, 0.90)	0.89 (0.86, 0.91)
2–<4	1.02 (1.00, 1.04)	1.04 (1.01, 1.06)	2–<4	0.97 (0.95, 1.00)	1.00 (0.97, 1.03)	2–<4	0.91 (0.89, 0.94)	0.94 (0.91, 0.96)
4–<6	0.92 (0.91, 0.94)	0.94 (0.92, 0.96)	4–<6	0.87 (0.85, 0.89)	0.89 (0.87, 0.91)	4–<6	0.88 (0.85, 0.90)	0.89 (0.87, 0.91)
6–<8	0.91 (0.90, 0.93)	0.94 (0.92, 0.95)	6–<8	0.88 (0.86, 0.90)	0.90 (0.88, 0.92)	6–<8	0.88 (0.86, 0.90)	0.89 (0.86, 0.91)
8–<10	0.88 (0.87, 0.90)	0.90 (0.89, 0.91)	8–<10	0.91 (0.89, 0.93)	0.92 (0.90, 0.94)	8–<10	0.90 (0.88, 0.92)	0.91 (0.88, 0.93)
10–<12	0.94 (0.92, 0.95)	0.95 (0.94, 0.97)	10–<12	0.98 (0.96, 1.00)	0.99 (0.97, 1.01)	10–<12	0.98 (0.96, 1.01)	0.98 (0.96, 1.01)
12–<14	0.93 (0.92, 0.95)	0.94 (0.93, 0.96)	12–<14	0.95 (0.93, 0.97)	0.95 (0.93, 0.98)	12–<14	1.02 (0.99, 1.05)	1.02 (0.99, 1.05)
14–<16	0.96 (0.94, 0.97)	0.96 (0.94, 0.97)	14–<16	0.98 (0.96, 1.01)	0.97 (0.95, 0.99)	14–<16	1.07 (1.04, 1.10)	1.05 (1.02, 1.08)
16–<18	1.02 (1.00, 1.04)	1.01 (0.99, 1.03)	16–<18	1.08 (1.05, 1.11)	1.06 (1.03, 1.09)	16–<18	1.19 (1.15, 1.23)	1.16 (1.12, 1.20)
18–<20	1.04 (1.02, 1.06)	1.02 (1.00, 1.04)	18–<20	1.09 (1.06, 1.12)	1.07 (1.03, 1.10)	18–<20	1.18 (1.14, 1.23)	1.15 (1.10, 1.19)
20–<22	1.09 (1.07, 1.12)	1.06 (1.04, 1.09)	≥20	1.38 (1.36, 1.41)	1.31 (1.29, 1.34)	≥20	1.55 (1.51, 1.58)	1.47 (1.44, 1.51)
22–<24	1.08 (1.06, 1.11)	1.04 (1.01, 1.07)						
24–<26	1.20 (1.17, 1.24)	1.14 (1.11, 1.18)						
26–<28	1.23 (1.19, 1.27)	1.16 (1.12, 1.20)						
28–<30	1.33 (1.27, 1.38)	1.24 (1.19, 1.29)						
≥30	1.69 (1.65, 1.73)	1.55 (1.51, 1.60)						

Abbreviations: GWG, gestational weight gain; COR, crude odds ratio; CI, confidence interval; AOR, adjusted odds ratio. [a]Adjusted for age (<25/25–34/≥35 years, categorical), race, education, marital status, smoking before or during pregnancy, parity, sex of infant, place of birth, and type of health insurance.

© 2021 Wang L et al. *JAMA Network Open.*

eTable 16. Associations Between Gestational Weight Gain and Infant Mortality in 2011–2015 in the US, Excluding Infants With Neural Tube Defects, by Prepregnancy Body Mass Index (N=15,756,060)

Underweight (n=628,788)			Normal weight (n=7,408,418)			Overweight (n=3,986,817)		
**GWG, kg**	**COR (95% CI)**	**AOR (95% CI)[a]**	**GWG, kg**	**COR (95% CI)**	**AOR (95% CI)[a]**	**GWG, kg**	**COR (95% CI)**	**AOR (95% CI)[a]**
			<0	7.67 (6.92, 8.50)	6.13 (5.50, 6.82)	<0	3.82 (3.51, 4.16)	3.31 (3.03, 3.62)
			0–<2	5.85 (5.48, 6.25)	4.61 (4.29, 4.95)	0–<2	2.85 (2.67, 3.04)	2.48 (2.31, 2.66)
			2–<4	4.55 (4.25, 4.88)	3.73 (3.46, 4.01)	2–<4	2.20 (2.04, 2.37)	2.03 (1.88, 2.20)
			4–<6	2.88 (2.72, 3.05)	2.48 (2.33, 2.63)	4–<6	1.54 (1.44, 1.65)	1.45 (1.35, 1.56)
<8	4.36 (3.94, 4.83)	4.03 (3.61, 4.49)	6–<8	2.09 (1.99, 2.19)	1.90 (1.81, 2.00)	6–<8	1.35 (1.27, 1.43)	1.33 (1.25, 1.41)
8–<10	1.48 (1.30, 1.69)	1.54 (1.34, 1.77)	8–<10	1.27 (1.21, 1.32)	1.23 (1.17, 1.29)	8–<10	0.99 (0.93, 1.04)	0.99 (0.93, 1.05)
10–<12	1.02 (0.90, 1.15)	1.09 (0.96, 1.24)	10–<12	0.93 (0.89, 0.97)	0.96 (0.91, 1.00)	10–<12	0.81 (0.77, 0.86)	0.85 (0.80, 0.90)
12–<14	0.73 (0.64, 0.83)	0.80 (0.70, 0.91)	12–<14	0.72 (0.69, 0.75)	0.78 (0.74, 0.81)	12–<14	0.73 (0.69, 0.77)	0.76 (0.72, 0.81)
14–<16	0.64 (0.56, 0.73)	0.66 (0.57, 0.76)	14–<16	0.65 (0.62, 0.68)	0.70 (0.67, 0.74)	14–<16	0.67 (0.63, 0.71)	0.71 (0.67, 0.76)
16–<18	0.59 (0.50, 0.69)	0.59 (0.50, 0.70)	16–<18	0.65 (0.62, 0.69)	0.70 (0.66, 0.74)	16–<18	0.70 (0.65, 0.75)	0.73 (0.67, 0.78)
18–<20	0.70 (0.60, 0.82)	0.71 (0.60, 0.84)	18–<20	0.66 (0.62, 0.70)	0.68 (0.65, 0.72)	18–<20	0.73 (0.68, 0.78)	0.74 (0.69, 0.80)
20–<22	0.52 (0.42, 0.65)	0.49 (0.39, 0.62)	20–<22	0.69 (0.65, 0.74)	0.70 (0.65, 0.74)	20–<22	0.72 (0.66, 0.78)	0.73 (0.67, 0.80)
22–<24	0.75 (0.59, 0.95)	0.70 (0.55, 0.90)	22–<24	0.72 (0.67, 0.78)	0.69 (0.64, 0.75)	22–<24	0.85 (0.77, 0.93)	0.85 (0.77, 0.94)
≥24	1.17 (1.01, 1.35)	0.99 (0.85, 1.15)	24–<26	0.84 (0.77, 0.92)	0.77 (0.70, 0.85)	24–<26	0.80 (0.72, 0.90)	0.80 (0.71, 0.90)
			26–<28	0.89 (0.80, 0.99)	0.80 (0.72, 0.90)	26–<28	0.78 (0.68, 0.89)	0.75 (0.65, 0.86)
			28–<30	1.01 (0.88, 1.15)	0.89 (0.78, 1.03)	28–<30	1.04 (0.89, 1.21)	0.98 (0.84, 1.15)
			≥30	1.60 (1.48, 1.73)	1.33 (1.23, 1.44)	≥30	1.46 (1.33, 1.60)	1.29 (1.17, 1.42)
**Obesity class 1 (n=2,074,563)**			**Obesity class 2 (n=980,215)**			**Obesity class 3 (n=677,259)**		
**GWG, kg**	**COR (95% CI)**	**AOR (95% CI)[a]**	**GWG, kg**	**COR (95% CI)**	**AOR (95% CI)[a]**	**GWG, kg**	**COR (95% CI)**	**AOR (95% CI)[a]**
<0	2.75 (2.54, 2.98)	2.52 (2.31, 2.74)	<0	2.12 (1.94, 2.32)	2.12 (1.93, 2.32)	<0	1.57 (1.44, 1.70)	1.58 (1.45, 1.73)
0–<2	2.17 (2.03, 2.33)	2.04 (1.89, 2.20)	0–<2	1.63 (1.49, 1.78)	1.53 (1.39, 1.69)	0–<2	1.41 (1.28, 1.55)	1.40 (1.27, 1.54)
2–<4	1.39 (1.28, 1.52)	1.38 (1.25, 1.51)	2–<4	1.19 (1.07, 1.33)	1.24 (1.11, 1.39)	2–<4	0.92 (0.82, 1.04)	0.98 (0.86, 1.11)
4–<6	1.17 (1.09, 1.27)	1.17 (1.08, 1.28)	4–<6	0.97 (0.88, 1.08)	1.00 (0.90, 1.11)	4–<6	0.90 (0.80, 1.00)	0.92 (0.82, 1.03)
6–<8	1.05 (0.98, 1.13)	1.05 (0.97, 1.13)	6–<8	0.85 (0.77, 0.94)	0.86 (0.78, 0.96)	6–<8	0.81 (0.73, 0.91)	0.83 (0.74, 0.93)
8–<10	0.80 (0.74, 0.86)	0.81 (0.75, 0.88)	8–<10	0.76 (0.68, 0.84)	0.76 (0.68, 0.85)	8–<10	0.81 (0.73, 0.91)	0.82 (0.73, 0.93)
10–<12	0.74 (0.69, 0.80)	0.78 (0.72, 0.84)	10–<12	0.72 (0.65, 0.81)	0.75 (0.67, 0.84)	10–<12	0.72 (0.64, 0.82)	0.73 (0.64, 0.84)
12–<14	0.70 (0.65, 0.76)	0.74 (0.68, 0.80)	12–<14	0.75 (0.67, 0.84)	0.76 (0.68, 0.85)	12–<14	0.81 (0.71, 0.92)	0.80 (0.70, 0.92)
14–<16	0.69 (0.63, 0.75)	0.71 (0.65, 0.78)	14–<16	0.78 (0.69, 0.88)	0.78 (0.69, 0.89)	14–<16	0.92 (0.80, 1.06)	0.93 (0.80, 1.07)
16–<18	0.73 (0.66, 0.80)	0.76 (0.69, 0.84)	16–<18	0.76 (0.66, 0.88)	0.75 (0.65, 0.88)	16–<18	0.68 (0.57, 0.82)	0.68 (0.56, 0.83)
18–<20	0.75 (0.68, 0.83)	0.76 (0.68, 0.85)	18–<20	0.84 (0.72, 0.97)	0.85 (0.73, 0.99)	18–<20	0.88 (0.74, 1.05)	0.82 (0.68, 0.99)
20–<22	0.79 (0.70, 0.89)	0.80 (0.71, 0.90)	≥20	1.10 (1.01, 1.21)	1.04 (0.94, 1.14)	≥20	1.24 (1.12, 1.37)	1.14 (1.02, 1.27)
22–<24	0.79 (0.69, 0.90)	0.77 (0.67, 0.89)						
24–<26	0.86 (0.74, 1.01)	0.80 (0.68, 0.95)						
26–<28	1.02 (0.86, 1.20)	0.97 (0.81, 1.15)						
28–<30	1.22 (1.00, 1.49)	1.15 (0.94, 1.42)						
≥30	1.74 (1.56, 1.94)	1.53 (1.36, 1.71)						

Abbreviations: GWG, gestational weight gain; COR, crude odds ratio; CI, confidence interval; AOR, adjusted odds ratio. [a]Adjusted for age (<25/25–34/≥35 years, categorical), race, education, marital status, smoking before or during pregnancy, parity, sex of infant, place of birth, and type of health insurance.

© 2021 Wang L et al. *JAMA Network Open.*

eTable 17. Associations Between Gestational Weight Gain and Infant Morbidity (Excluding NICU Admission) in 2011–2015 in the US, by Prepregnancy Body Mass Index (N=15,759,945)

**Underweight (n=628,929)**			**Normal weight (n=7,410,061)**			**Overweight (n=3,987,800)**		
**GWG, kg**	**COR (95% CI)**	**AOR (95% CI)[a]**	**GWG, kg**	**COR (95% CI)**	**AOR (95% CI)[a]**	**GWG, kg**	**COR (95% CI)**	**AOR (95% CI)[a]**
			<0	1.96 (1.86, 2.07)	1.84 (1.74, 1.95)	<0	1.42 (1.37, 1.48)	1.38 (1.33, 1.44)
			0–<2	1.51 (1.46, 1.56)	1.44 (1.39, 1.49)	0–<2	1.10 (1.07, 1.14)	1.09 (1.06, 1.12)
			2–<4	1.58 (1.53, 1.63)	1.54 (1.49, 1.59)	2–<4	1.11 (1.08, 1.15)	1.15 (1.12, 1.19)
			4–<6	1.25 (1.22, 1.28)	1.24 (1.21, 1.27)	4–<6	0.98 (0.95, 1.00)	1.01 (0.99, 1.04)
<8	1.63 (1.57, 1.71)	1.63 (1.56, 1.70)	6–<8	1.13 (1.11, 1.15)	1.15 (1.13, 1.17)	6–<8	0.94 (0.92, 0.96)	0.99 (0.97, 1.01)
8–<10	1.11 (1.06, 1.16)	1.15 (1.10, 1.20)	8–<10	0.96 (0.95, 0.97)	0.99 (0.98, 1.00)	8–<10	0.88 (0.87, 0.90)	0.92 (0.91, 0.94)
10–<12	0.99 (0.96, 1.03)	1.04 (1.00, 1.08)	10–<12	0.92 (0.91, 0.93)	0.95 (0.94, 0.96)	10–<12	0.90 (0.88, 0.91)	0.93 (0.92, 0.95)
12–<14	0.89 (0.86, 0.92)	0.94 (0.90, 0.97)	12–<14	0.89 (0.88, 0.90)	0.93 (0.92, 0.94)	12–<14	0.90 (0.89, 0.92)	0.93 (0.91, 0.94)
14–<16	0.85 (0.82, 0.88)	0.88 (0.85, 0.92)	14–<16	0.89 (0.88, 0.90)	0.92 (0.91, 0.93)	14–<16	0.93 (0.92, 0.94)	0.93 (0.92, 0.95)
16–<18	0.90 (0.87, 0.94)	0.91 (0.88, 0.95)	16–<18	0.95 (0.94, 0.96)	0.96 (0.95, 0.97)	16–<18	1.01 (0.99, 1.02)	1.00 (0.98, 1.02)
18–<20	0.93 (0.89, 0.97)	0.90 (0.86, 0.94)	18–<20	0.99 (0.98, 1.00)	0.98 (0.96, 0.99)	18–<20	1.01 (0.99, 1.03)	0.99 (0.97, 1.01)
20–<22	0.97 (0.92, 1.01)	0.91 (0.86, 0.95)	20–<22	1.01 (1.00, 1.03)	0.98 (0.97, 1.00)	20–<22	1.06 (1.04, 1.08)	1.01 (0.99, 1.04)
22–<24	1.00 (0.94, 1.06)	0.92 (0.87, 0.98)	22–<24	1.07 (1.05, 1.09)	1.01 (0.99, 1.03)	22–<24	1.09 (1.06, 1.11)	1.03 (1.00, 1.05)
≥24	1.19 (1.14, 1.24)	1.06 (1.02, 1.11)	24–<26	1.16 (1.14, 1.19)	1.07 (1.05, 1.09)	24–<26	1.19 (1.16, 1.22)	1.10 (1.07, 1.13)
			26–<28	1.18 (1.15, 1.20)	1.06 (1.04, 1.09)	26–<28	1.20 (1.17, 1.24)	1.11 (1.07, 1.14)
			28–<30	1.29 (1.25, 1.33)	1.15 (1.11, 1.18)	28–<30	1.30 (1.25, 1.35)	1.17 (1.12, 1.22)
			≥30	1.47 (1.44, 1.50)	1.27 (1.24, 1.29)	≥30	1.52 (1.48, 1.56)	1.35 (1.31, 1.38)
**Obesity class 1 (n=2,075,150)**			**Obesity class 2 (n=980,500)**			**Obesity class 3 (n=677,505)**		
**GWG, kg**	**COR (95% CI)**	**AOR (95% CI)[a]**	**GWG, kg**	**COR (95% CI)**	**AOR (95% CI)[a]**	**GWG, kg**	**COR (95% CI)**	**AOR (95% CI)[a]**
<0	1.18 (1.14, 1.22)	1.19 (1.15, 1.23)	<0	1.06 (1.02, 1.09)	1.08 (1.04, 1.12)	<0	0.95 (0.92, 0.98)	0.95 (0.92, 0.99)
0–<2	0.96 (0.94, 0.99)	0.97 (0.94, 1.00)	0–<2	0.94 (0.90, 0.97)	0.95 (0.92, 0.98)	0–<2	0.93 (0.90, 0.96)	0.94 (0.91, 0.97)
2–<4	1.00 (0.97, 1.03)	1.04 (1.00, 1.07)	2–<4	0.98 (0.95, 1.02)	1.02 (0.99, 1.06)	2–<4	0.94 (0.91, 0.98)	0.97 (0.93, 1.01)
4–<6	0.91 (0.88, 0.93)	0.94 (0.92, 0.97)	4–<6	0.88 (0.86, 0.91)	0.91 (0.88, 0.94)	4–<6	0.92 (0.89, 0.95)	0.93 (0.90, 0.96)
6–<8	0.90 (0.88, 0.92)	0.94 (0.92, 0.96)	6–<8	0.91 (0.88, 0.94)	0.93 (0.91, 0.96)	6–<8	0.90 (0.87, 0.93)	0.91 (0.88, 0.94)
8–<10	0.88 (0.86, 0.90)	0.91 (0.89, 0.93)	8–<10	0.91 (0.88, 0.93)	0.92 (0.90, 0.95)	8–<10	0.90 (0.87, 0.93)	0.91 (0.88, 0.94)
10–<12	0.92 (0.90, 0.94)	0.95 (0.93, 0.97)	10–<12	0.97 (0.94, 1.00)	0.97 (0.94, 1.00)	10–<12	0.96 (0.93, 0.99)	0.96 (0.93, 1.00)
12–<14	0.94 (0.92, 0.96)	0.95 (0.93, 0.97)	12–<14	0.96 (0.93, 0.99)	0.96 (0.93, 0.99)	12–<14	1.03 (1.00, 1.07)	1.03 (1.00, 1.07)
14–<16	0.97 (0.95, 0.99)	0.96 (0.94, 0.98)	14–<16	1.01 (0.97, 1.04)	0.98 (0.95, 1.02)	14–<16	1.03 (0.99, 1.08)	1.01 (0.97, 1.06)
16–<18	1.03 (1.01, 1.06)	1.01 (0.98, 1.04)	16–<18	1.03 (0.99, 1.07)	1.00 (0.97, 1.04)	16–<18	1.14 (1.09, 1.20)	1.11 (1.06, 1.17)
18–<20	1.08 (1.05, 1.10)	1.03 (1.01, 1.06)	18–<20	1.10 (1.05, 1.14)	1.05 (1.01, 1.10)	18–<20	1.14 (1.08, 1.20)	1.09 (1.04, 1.15)
20–<22	1.12 (1.09, 1.16)	1.07 (1.03, 1.10)	≥20	1.31 (1.28, 1.35)	1.23 (1.20, 1.26)	≥20	1.42 (1.38, 1.47)	1.35 (1.31, 1.40)
22–<24	1.14 (1.10, 1.18)	1.07 (1.03, 1.10)						
24–<26	1.22 (1.17, 1.27)	1.13 (1.09, 1.18)						
26–<28	1.24 (1.19, 1.30)	1.13 (1.08, 1.19)						
28–<30	1.27 (1.20, 1.35)	1.15 (1.09, 1.22)						
≥30	1.59 (1.54, 1.65)	1.44 (1.39, 1.49)						

Abbreviations: GWG, gestational weight gain; COR, crude odds ratio; CI, confidence interval; AOR, adjusted odds ratio. Note: “Infant morbidity (excluding NICU admission)” denoted any presence of assisted ventilation, surfactant therapy, antibiotic therapy, or seizures, regardless of admission to the neonatal intensive care unit. [a]Adjusted for age (<25/25–34/≥35 years, categorical), race, education, marital status, smoking before or during pregnancy, parity, sex of infant, place of birth, and type of health insurance.

© 2021 Wang L et al. *JAMA Network Open.*
